# Surface‐Exposed Pd Nanocluster Confined within a Ring‐Shaped Polyoxometalate for Selective Hydrogenation

**DOI:** 10.1002/advs.202509418

**Published:** 2025-07-28

**Authors:** Rui Xi, Kentaro Yonesato, Takafumi Yatabe, Yoshihiro Koizumi, Soichi Kikkawa, Seiji Yamazoe, Koji Harano, Kazuya Yamaguchi, Kosuke Suzuki

**Affiliations:** ^1^ Department of Applied Chemistry School of Engineering The University of Tokyo 7‐3‐1 Hongo, Bunkyo‐ku Tokyo 113‐8656 Japan; ^2^ Department of Chemistry Graduate School of Science Tokyo Metropolitan University 1‐1 Minami Osawa, Hachioji Tokyo 192‐0397 Japan; ^3^ Center for Basic Research on Materials National Institute for Materials Science (NIMS) 1‐1 Namiki, Tsukuba Ibaraki 305‐0044 Japan; ^4^ Research Center for Autonomous Systems Materialogy (ASMat) Institute of Integrated Research Institute of Science Tokyo 4259 Nagatsuda‐cho, Midori‐ku Yokohama Kanagawa 226‐8501 Japan; ^5^ Department of Advanced Materials Science Graduate School of Frontier Sciences The University of Tokyo 5‐1‐5 Kashiwanoha, Kashiwa Chiba 277‐8561 Japan

**Keywords:** heterogeneous catalysis, palladium nanoclusters, polyoxometalates, selective hydrogenations

## Abstract

Developing efficient catalysts for selective hydrogenation of molecules bearing multiple reducible functional groups remains a major challenge. Palladium (Pd) nanoclusters are promising candidates owing to their strong H_2_ activation ability, broad substrate compatibility, and unique surface properties. However, the controlled synthesis of small Pd nanoclusters with accessible, coordinatively unsaturated active sites remains difficult as they are prone to aggregation. In this study, a strategy is presented to fabricate surface‐exposed Pd nanoclusters confined within a ring‐shaped polyoxometalate (POM) via a mild solid‐state reduction process (1 atm H_2_, ≈25 °C). The resulting Pd nanocluster exhibits exceptional chemoselectivity in the hydrogenation of multifunctional substrates by preferentially adsorbing C═C and C≡C bonds on its discrete, exposed Pd surface with a well‐defined coordination environment. Importantly, the rigid POM framework considerably stabilizes Pd nanocluster, enabling excellent reusability over multiple catalytic cycles. This study demonstrates a molecular templating approach for constructing robust and chemoselective metal nanocluster catalysts, offering new opportunities in the design of hydrogenation systems.

## Graphical abstract

A small Pd nanocluster is successfully prepared within a ring‐shaped polyoxometalate via a mild solid‐state reduction process. The resulting surface‐exposed Pd nanocluster functions as a robust heterogeneous reusable catalyst, enabling highly chemoselective hydrogenation of substrates containing multiple reducible functional groups via selective adsorption on its discrete Pd surface.

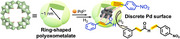

## 1 Introduction

**Figure 1:**
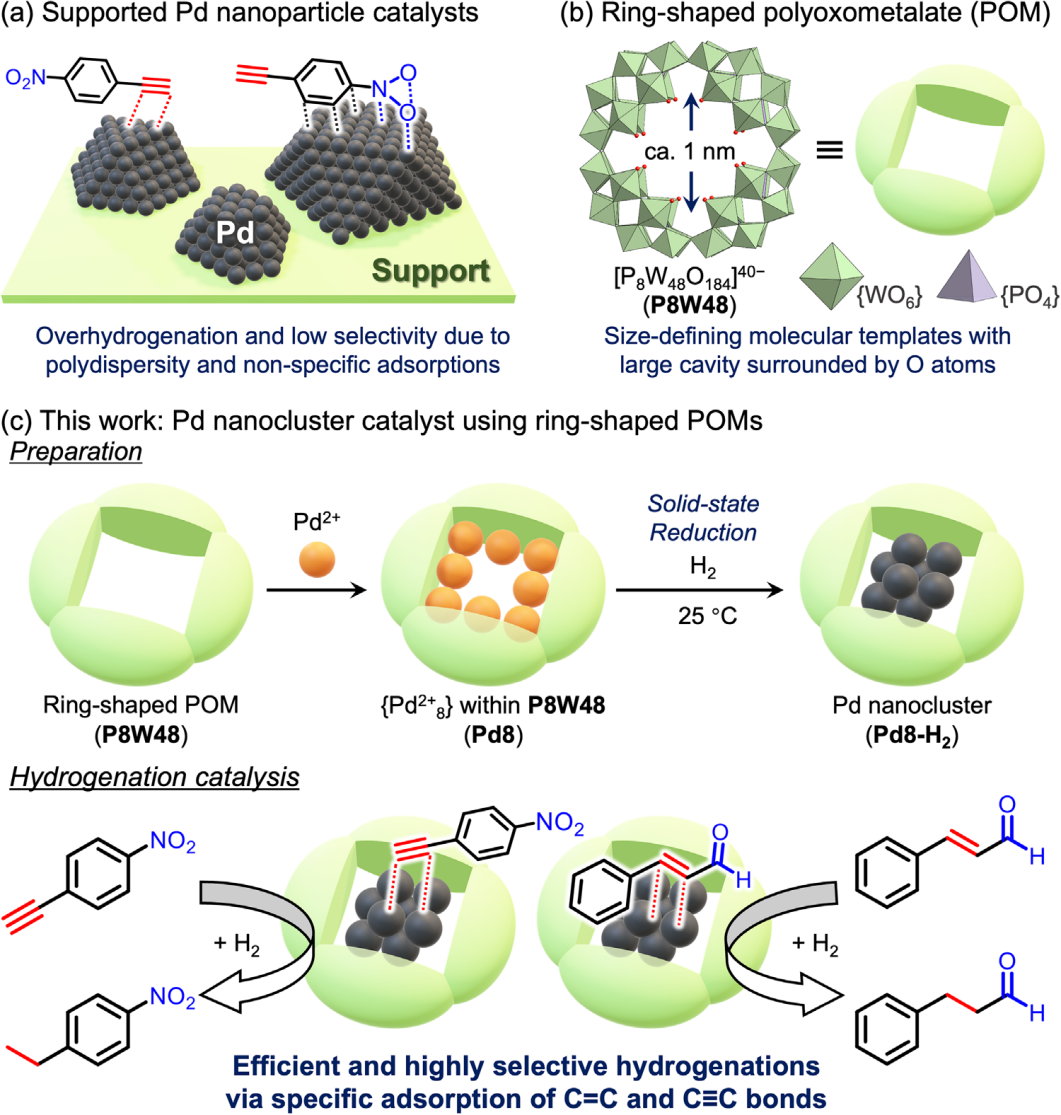
Schematic representation of the proposed synthesis method: a) supported Pd nanoparticle catalyst for hydrogenation reactions, b) ring‐shaped polyoxometalate ([P_8_W_48_O_184_]^40−^, P8W48), and c) preparation of surface‐exposed Pd nanocluster catalyst using P8W48 and its selective hydrogenation ability through selective adsorption of functional groups.

Selective hydrogenation of molecules containing multiple reducible functional groups is a cornerstone of modern chemical manufacturing, playing a crucial role in the production of petrochemicals, pharmaceuticals, and fine chemicals.^[^ ^1^ ^]^ However, achieving high chemoselectivity in such transformations is still a longstanding challenge. Undesired overhydrogenation and side reactions reduce product yields and complicate downstream purification, thereby increasing energy consumption and environmental burden. Therefore, the development of heterogeneous catalysts that combine high activity with precise chemoselectivity is of central importance for sustainable catalysis.^[^ ^2^ ^]^ Palladium (Pd) nanoparticles on supports are widely employed catalysts in hydrogenation reactions owing to their strong ability to activate molecular hydrogen (H_2_) and their affinity for various organic substrates, enabling high activity under mild conditions.^[^ ^3^, ^4^ ^]^ Nevertheless, achieving high chemoselectivity remains difficult. This is largely attributed to the polydispersity and low stability of Pd nanoparticles, which often results in aggregation under reaction conditions. Therefore, nonspecific adsorption of functional groups on extended Pd surfaces frequently leads to overhydrogenation and poor selectivity (**Figure** **1**a). To overcome these issues, various strategies have been developed.^[^ ^5^ ^]^ These include molecular poisoning (e.g., CO, thiols, and amines) to block adjacent Pd atoms,^[^ ^6^ ^]^ incorporation of secondary metals to deactivate unselective Pd sites,^[^ ^7^ ^]^ and alloying to electronically modulate or geometrically isolate Pd atoms.^[^ ^7^ ^]^ A classic example is Lindlar's catalyst (Pd/CaCO_3_–Pb with quinoline), which employs secondary metal addition and molecular poisoning to achieve alkyne semihydrogenation.^[^ ^8^ ^]^ However, these approaches frequently suffer from a trade‐off: enhancing selectivity often comes at the expense of catalytic activity owing to excessive surface blocking.^[^ ^9^ ^]^

To overcome this limitation, an ideal catalyst should possess a well‐defined Pd architecture with coordinatively unsaturated active sites that are accessible and structurally stabilized against aggregation. Metal nanoclusters represent a promising platform in this context owing to their discrete electronic structures and tunable reactivity.^[^ ^10^, ^11^ ^]^ However, their high surface energy renders them thermodynamically unstable, often leading to aggregation or deactivation. Although stabilization with organic ligands can suppress aggregation,^[^ ^10^, ^11^, ^12^ ^]^ it often comes at the expense of catalytic activity by blocking active sites. Immobilization on solid supports can improve thermal stability and help to preserve coordinatively unsaturated metal sites, but achieving precise control over cluster size, structure, and electronic state remains challenging—especially for inherently unstable metal clusters.^[^ ^13^ ^]^ Therefore, the development of stable Pd nanocluster catalysts with well‐defined structures and accessible, coordinatively unsaturated active sites continues to be a key objective in catalyst design. Polyoxometalates (POMs), anionic metal‐oxide clusters with well‐defined structures and diverse properties,^[^ ^14^ ^]^ have emerged as promising molecular templates for constructing metal clusters.^[^ ^15^ ^]^ In particular, lacunary POMs, which contain vacant sites with reactive oxygen atoms, can coordinate metal ions in specific geometries, enabling electronic modulation and structural stabilization.^[^ ^16^, ^17^ ^]^ These features allow them to stabilize metal nanoclusters and modulate their electronic and catalytic properties.^[^ ^18^ ^]^ Among various POM architectures, the ring‐shaped [P_8_W_48_O_184_]^40−^ (P8W48), a tetramer of the hexavacant Dawson‐type [P_2_W_12_O_48_]^14−^, offers a unique cavity (≈1 nm in diameter) surrounded by multiple coordination sites, allowing selective encapsulation and stabilization of metal species.^[^ ^19^ ^]^ Our group has recently demonstrated that P8W48 can serve as a versatile platform for synthesizing surface‐exposed Ag nanoclusters,^[^ ^20^ ^]^ Au–Ag alloy nanoclusters,^[^ ^21^ ^]^ and Cu nanoclusters^[^ ^22^ ^]^ with active surfaces and excellent catalytic performance and stability.

Herein, we report the synthesis of a small, surface‐exposed Pd nanocluster confined within the P8W48 framework and its application as a chemoselective heterogeneous hydrogenation catalyst (Figure 1b). The nanocluster, denoted as Pd8‐H_2_, was prepared via mild solid‐state reduction of a P8W48‐incorporated Pd^2+^ precursor (Pd8) under 1 atm H_2_ at room temperature (≈25 °C). In situ X‐ray absorption spectroscopy (XAS) revealed a reversible interconversion between the Pd^2^⁺ complex and the Pd^0^ nanocluster. Notably, Pd8‐H_2_ features a surface‐exposed, discrete Pd sites with well‐defined coordination environment, enabling efficient and highly selective hydrogenation of substrates bearing multiple reducible groups—for example, the selective conversion of cinnamaldehyde (**1** **a**) to hydrocinnamaldehyde (**2** **a**), and 1‐ethynyl‐4‐nitrobenzene (**1** **e**) to 4‐ethylnitrobenzene (**2** **e**). Remarkably, the structural rigidity of the P8W48 framework provides exceptional thermal and chemical stability, enabling reusability over multiple catalytic cycles. These findings highlight the potential of POM‐based molecular templating to achieve surface control and superior chemoselectivity, offering a powerful strategy for designing next‐generation nanocluster catalysts for diverse molecular transformations.

## 2 Results and Discussion

### 2.1 Synthesis of a Small Pd Nanocluster within P8W48

**Figure 2:**
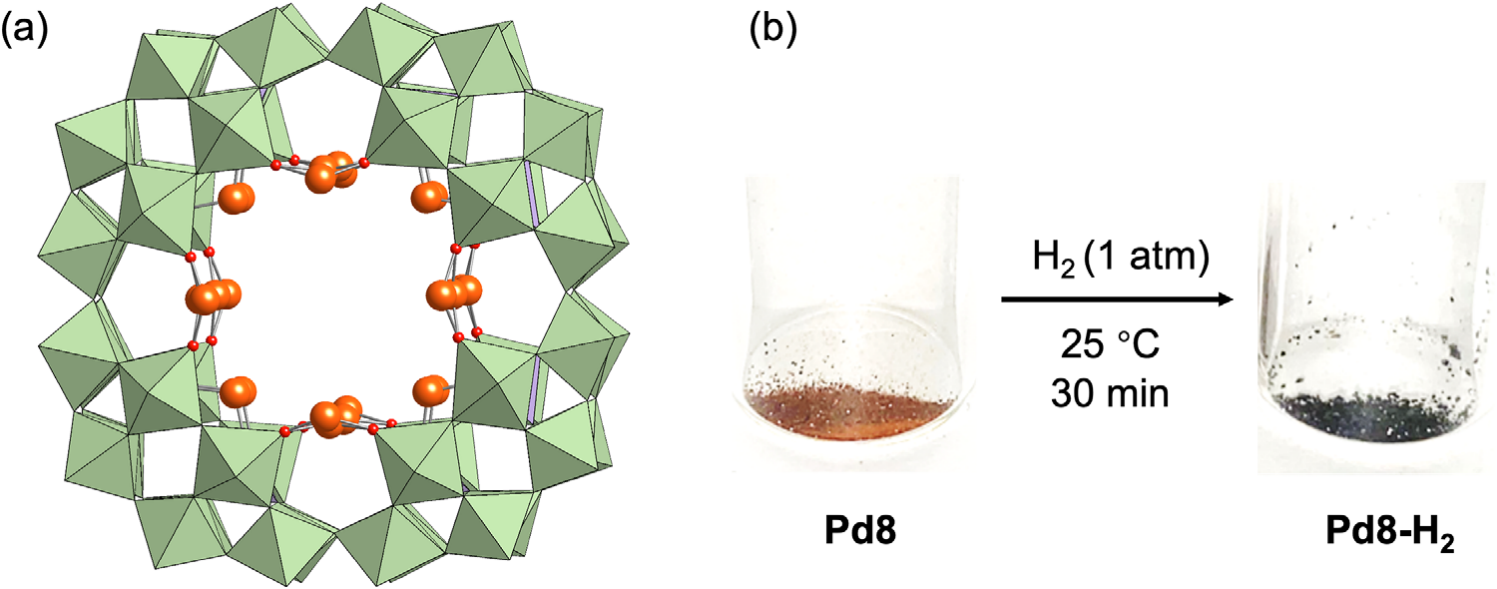
a) Anion structure of Pd8 (disordered Pd sites). b) Photographs of Pd8 before and after H_2_ treatment at room temperature (≈25 °C) for 30 min.

The highly reactive O atoms within the P8W48 cavity act as anchoring sites for metal ion coordination.^[^ ^19^, ^20^, ^21^, ^22^ ^]^ To incorporate Pd^2+^ ions, the tetra‐*n*‐butylammonium (TBA^+^) salt of P8W48 (TBA‐P8W48) was reacted with Pd acetate in acetone at room temperature (≈25 °C) over 3 d (see Supporting Information for experimental details). Subsequent addition of diethyl ether yielded an orange crystalline product, denoted Pd8, in 37% yield (Figure S1a, Supporting Information). Elemental analysis confirmed the incorporation of eight Pd^2+^ ions with the P8W48 framework. Single‐crystal X‐ray diffraction (SCXRD) further revealed coordination of Pd^2+^ ions to the inner O atoms of the cavity, although positional disorder was evident (**Figures** **2**a and S2a, and Table S1, Supporting Information).

Initial attempts to synthesize Pd nanoclusters within P8W48 involved reducing Pd8 in various organic solvents, such as acetone, acetonitrile, and *N*,*N*‐dimethylformamide, using H_2_ or TBA borohydride (TBABH_4_) as reducing agents. However, these solution‐phase reductions consistently yielded black Pd precipitates (Figure S3, Supporting Information). Elemental analysis of the resulting crystals revealed that only two Pd atoms per P8W48 unit remained, indicating that the reduced Pd species had been eluted from the framework followed by aggregation in the solution. To circumvent this issue, a solid‐state reduction approach was employed.^[^ ^22^ ^]^ Exposure of Pd8 to H_2_ gas (1 atm) at room temperature (≈25 °C) led to a rapid color change from orange to blackish (Figure 2b), indicative of Pd^2+^ reduction to Pd^0^. Recrystallization of the resulting blackish solid (Pd8‐H_2_) in a mixture of acetone and diethyl ether under an Ar atmosphere yielded blackish single crystals. Elemental analysis confirmed the retention of all eight Pd atoms within the P8W48 framework, demonstrating that solid‐state reduction effectively suppressed Pd elution and aggregation. These findings suggest that Pd nanocluster formation occurred in situ within the P8W48 cavity during the solid‐state reduction, whereas solution‐phase reduction likely facilitated Pd detachment through solvent interactions, leading to uncontrolled aggregation. Thus, solid‐state reduction was essential for the controlled synthesis of Pd nanoclusters confined within the P8W48 framework.

**Figure 3:**
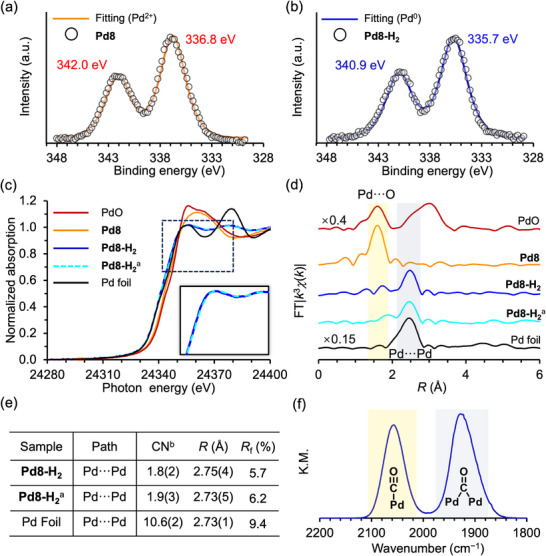
XPS spectra of a) Pd8 and b) Pd8‐H_2_. c) Pd K‐edge XANES spectra, d) *R*‐space Pd K‐edge EXAFS spectra, and e) curve−fitting results of Pd8, Pd8‐H_2_, Pd8‐H_2_^a^, and Pd foil. Fitting parameters of the Pd–Pd interactions (*R* range = 2.10–2.86 Å and back *k* range = 3–14 Å^−1^; Pd foil, *R* range = 1.90–2.85 Å). ^a^Pd8‐H_2_ after recrystallization. ^b^Coordination number. f) CO‐DRIFTS spectrum of Pd8‐H_2_.

X‐ray photoelectron spectroscopy (XPS) of Pd8 revealed two distinct peaks at 336.8 eV (Pd 3d_5/2_) and 342.0 eV (Pd 3d_3/2_), consistent with the presence of Pd^2+^ species (**Figure** **3**a). Following H_2_ treatment, the corresponding peaks in Pd8‐H_2_ shifted to lower binding energies—335.7 eV (Pd 3*d*_5/2_) and 340.9 eV (Pd 3*d*_3/2_)—indicating the reduction of Pd^2+^ to Pd^0^ (Figure 3b). This assignment was further corroborated by Pd K‐edge X‐ray absorption near‐edge structure (XANES) analysis. The Pd8‐H_2_ spectrum exhibited a diminished white‐line intensity (≈24356 eV) and a negative edge energy shift relative to Pd8, closely resembling that of Pd foil (Figure 3c).

Infrared (IR) spectra of P8W48, Pd8, and Pd8‐H_2_ in the 1200–500 cm^−1^ range confirmed that the P8W48 framework remained structurally intact following H_2_ reduction (Figure S4, Supporting Information). Extended X‐ray absorption fine structure (EXAFS) analysis provided further insights into the local coordination environment of the Pd species. The *R*‐space EXAFS spectra exhibited a transition from predominant Pd–O coordination in Pd8 to Pd–Pd interactions in Pd8‐H_2_ (Figure 3d), consistent with the formation of metallic Pd clusters. Curve‐fitting analysis yielded a Pd–Pd coordination number (CN) of 1.8 ± 0.2 for Pd8‐H_2_—significantly lower than that of bulk Pd foil (10.6 ± 0.2; Figure 3e) and commercial Pd nanoparticles supported on carbon (Pd/C, 5.1 ± 0.2; Table S2, Supporting Information)—supporting the formation of a small Pd nanocluster. Notably, the XANES and EXAFS spectra of recrystallized Pd8‐H_2_ (CN = 1.9 ± 0.3) closely matched those of the as‐reduced sample, indicating that both the electronic state and cluster size were preserved during recrystallization under an Ar atmosphere (Figure 3c–e). SCXRD analysis of Pd8‐H_2_ revealed electron density consistent with Pd atoms localized at the center of the P8W48 cavity. No significant electron density corresponding to Pd was detected outside the framework, further substantiating the confinement of the Pd nanocluster within the P8W48 cavity (Figure S2b and Table S1, Supporting Information).

**Figure 4:**
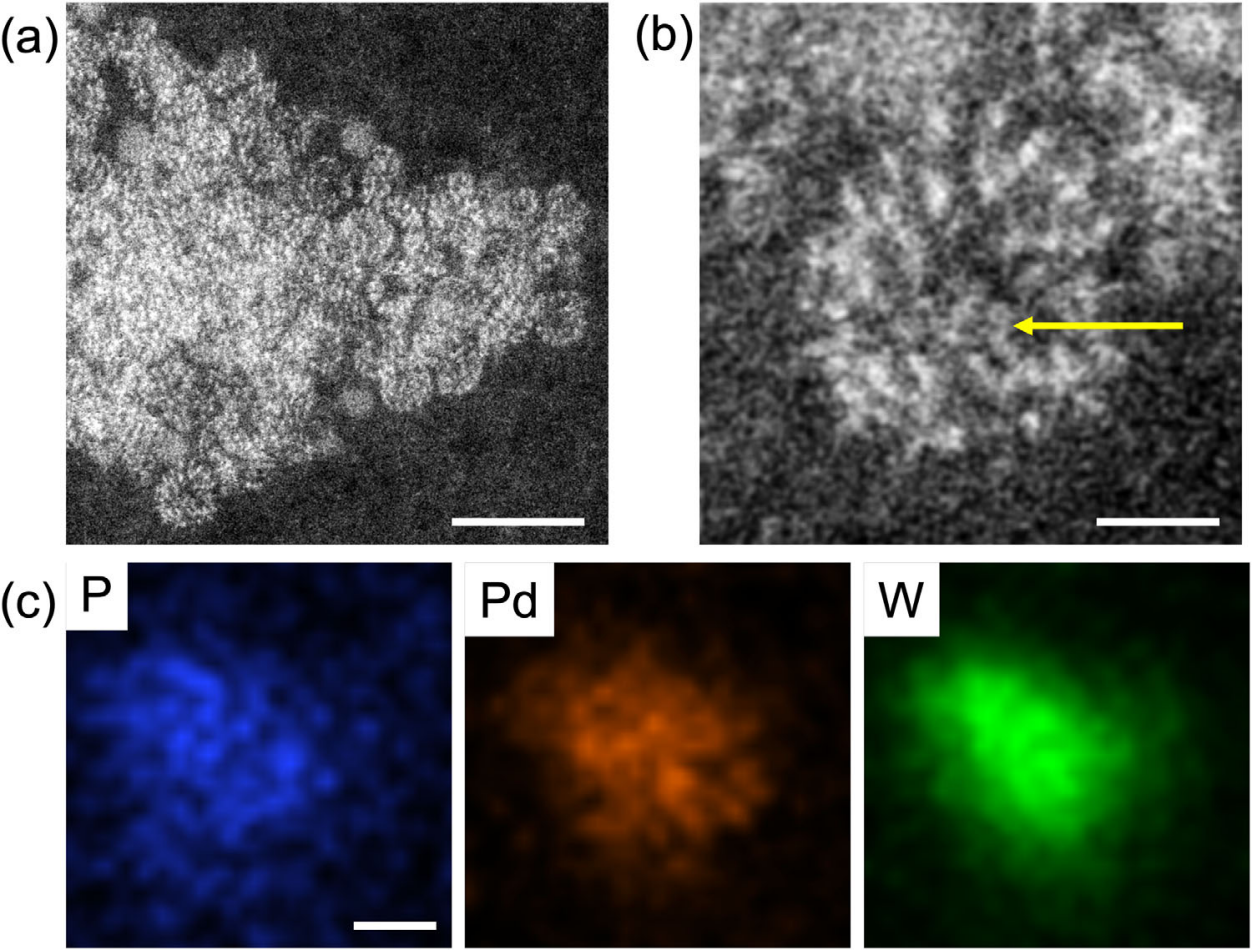
STEM images of Pd8‐H_2_. a) ADF‐STEM image of an aggregate of Pd8‐H_2_ particles on an amorphous carbon film. b) A magnified view of a single Pd8‐H_2_ particle, showing an encapsulated Pd cluster in the P8W48 cavity (yellow arrow). c) EDS elemental maps of P, Pd, and W. Scale bars: 5 nm for a, 1 nm for b,c.

To further confirm the spatial confinement of Pd within the P8W48 cavity, atomic‐resolution scanning transmission electron microscopy (STEM) was performed. Although individual POM molecules are typically challenging to visualize due to their sensitivity to electron beam damage, careful control of beam intensity during annular dark‐field (ADF) STEM imaging enabled the observation of a bright contrast feature located within the central ring of the P8W48 framework (**Figures** **4**a,b and S5, Supporting Information). This contrast is attributed to a Pd species encapsulated within the cavity. Notably, such atomic‐resolution STEM imaging of an isolated POM molecule—without the aid of encapsulation in carbon nanotubes^[^ ^23^ ^]^—is exceedingly rare. To further validate the presence and localization of Pd, STEM–energy‐dispersive X‐ray spectroscopy (EDS) elemental mapping was conducted. Although the higher beam intensity required for EDS led to some degradation of the molecular structure, the elemental maps clearly showed co‐localization of Pd, P, and W within a ≈2 nm region (Figure 4c), providing strong evidence for the formation of a Pd nanocluster confined within the ring‐shaped P8W48 framework.

The surface structure of heterogeneous catalysts critically influences their catalytic behavior. Carbon monoxide (CO) is commonly employed as a probe molecule due to its strong adsorption on metal surfaces and its ability to yield distinct IR signatures that reflect local surface environments.^[^ ^24^ ^]^ Diffuse reflectance IR Fourier transform spectroscopy (DRIFTS) of Pd8‐H_2_ after CO exposure at room temperature revealed two sharp absorption bands at 2060 and 1930 cm^−1^, corresponding to linearly and bridge‐bonded CO on Pd, respectively (Figure 3f).^[^ ^24b^ ^]^ The dominance of the linear CO band indicates a high proportion of low‐coordination Pd surface sites, a hallmark of small Pd nanoclusters. Notably, the absence of a band near 1850 cm^−1^—typically associated with tri‐bonded CO species—suggests the lack of extended Pd surfaces, clearly distinguishing Pd8‐H_2_ from conventional Pd nanoparticles.^[^ ^24b,d,e^ ^]^ The sharpness and symmetry of the CO stretching bands further suggest a uniform distribution of surface‐active sites. These results collectively indicate that Pd8‐H_2_ possesses a discrete, surface‐exposed Pd nanocluster with a well‐defined coordination environment, potentially contributing to its unique catalytic properties.

### 2.2 Structural Transformation of Pd8 into Pd8‐H_2_

**Figure 5:**
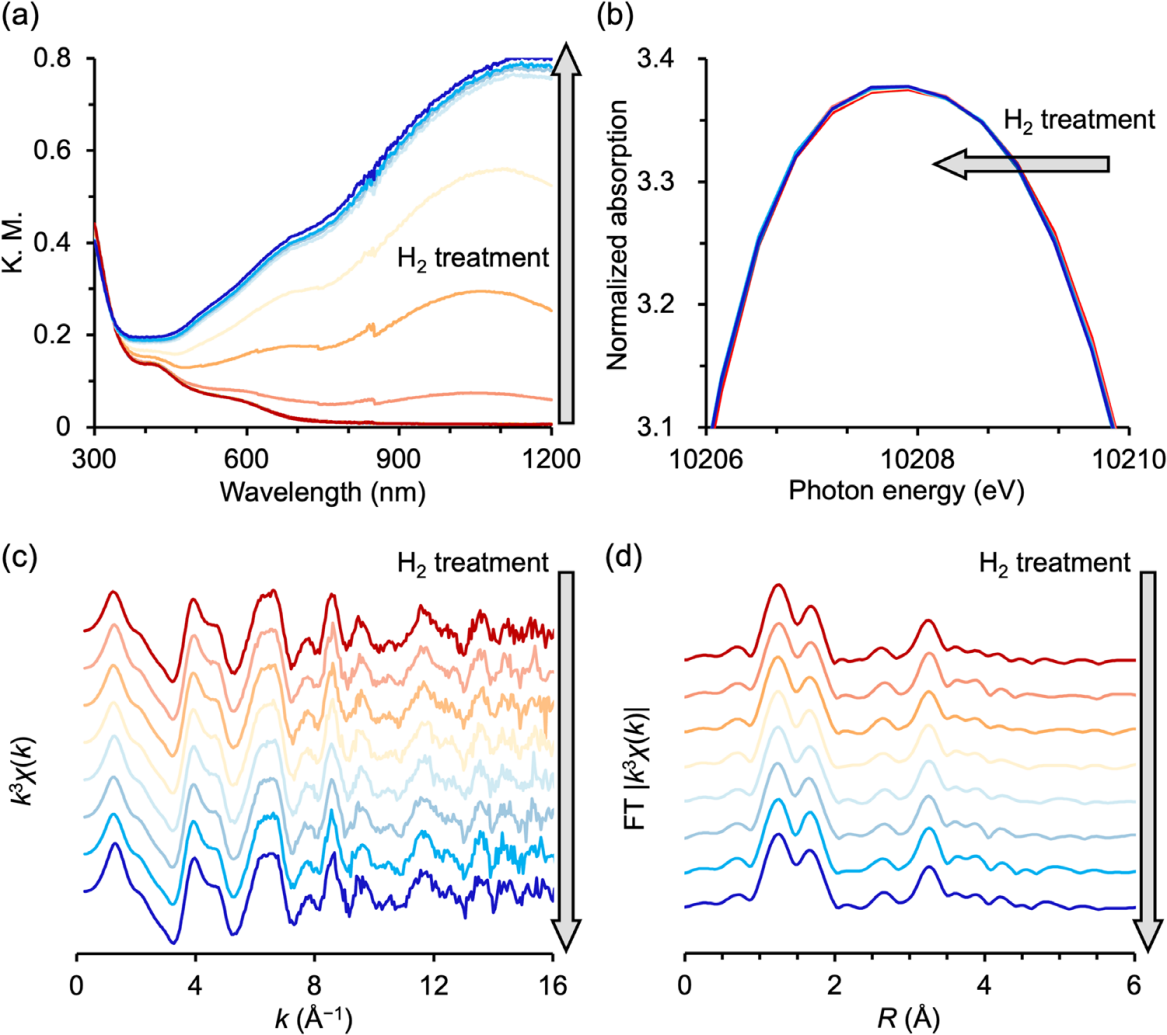
a) In situ diffuse reflectance solid‐state UV–vis spectra of Pd8 under H_2_ gas flow at 50 °C; the interval of each measurement was 2 min. b) In situ W L_3_‐edgeXANES spectra, c) in situ *k* ^3^‐weighted *k*‐space, and d) *R‐*space W L_3_‐edge EXAFS spectra of Pd8 with a H_2_ flow at 50 °C; the interval of each measurement was 7 min.

The structural transformation of Pd8 into Pd8‐H_2_ under H_2_ was monitored using a range of in situ characterization techniques. Solid‐state ultraviolet–visible (UV–vis) spectra of Pd8 under H_2_ flow at 50 °C revealed gradually intensified broad absorption bands around 650 and 1000 nm, corresponding to W(VI)/W(V) intervalence charge transfer within the P8W48 framework (**Figure** **5**a). This behavior suggests partial reduction of W(VI) to W(V). Consistent with this, the W L_3_‐edge XANES spectra showed a slight shift of the white line (10209 eV) toward lower energy (Figure 5b), further supporting the partial reduction of W(VI). Importantly, both the W L_3_‐edge *k*‐space and *R*‐space EXAFS spectra exhibited no significant structural changes during the H_2_ treatment (Figure 5c,d), confirming the preservation of the P8W48 framework. These results indicate that P8W48 serves as a robust inorganic protective ligand, facilitating the formation of metal nanoclusters under reductive conditions.

**Figure 6:**
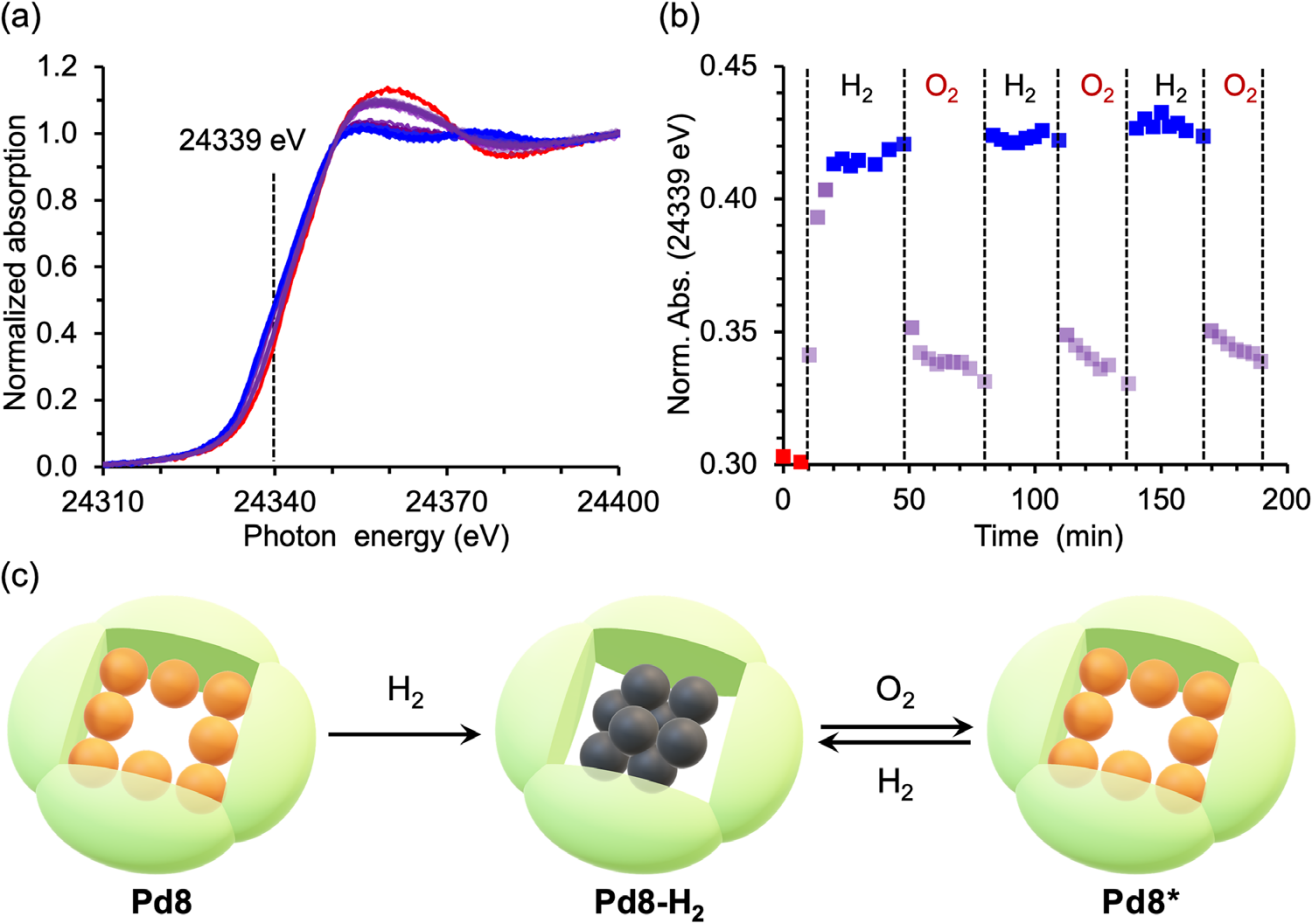
a) Schematic of reversible structural transformations. b) In situ Pd K‐edge XANES spectra of Pd8 under alternating H_2_ and O_2_ flow at 50 °C. c) Overall time profile of the normalized absorption intensity (Norm. Abs.) at energy in the pre‐edge region (24339 eV).

Interestingly, upon exposure to air at room temperature, the color of Pd8‐H_2_ gradually shifted from blackish to brown, and reverted to blackish upon H_2_ treatment (Figure S6, Supporting Information), indicating reversible changes in the oxidation state of the Pd species. To further investigate this behavior, in situ Pd K‐edge XAS studies were performed under alternating H_2_ and O_2_ flow at 50 °C. The Pd K‐edge XANES spectra revealed a time‐dependent decrease in white‐line intensity (≈24356 eV) and a corresponding increase in pre‐edge peak intensity (≈24340 eV), both of which reached completion within 10 min (**Figure** **6**a), indicating the rapid reduction of Pd^2+^ into Pd^0^. The alternating H_2_ and O_2_ flows demonstrated the reversible redox behavior of Pd. Notably, the XANES spectrum and white‐line intensity after O_2_ exposure slightly differed from those of the pristine Pd8, suggesting a subtle modification in the electronic states or coordination environments (Figure 6b). Despite repeated cycles, both Pd K‐edge XANES and EXAFS spectra of the final state remained unchanged (Figure S7, Supporting Information), confirming the stability of the reduced state and structural integrity of the Pd nanocluster (Figure 6c). Crystallographic analysis of the reoxidized product, Pd8* (obtained via exposing a Pd8‐H_2_ single crystal to O_2_), demonstrated the disappearance of central electron density and re‐coordination of all Pd atoms to O atoms within the P8W48 framework (Figure S2c and Table S1, Supporting Information). Although the Pd atoms in Pd8* were severely disordering over multiple positions, the analysis showed that the several Pd sites differed from those in the pristine Pd8, which accounting for the slight differences observed in the XANES spectra. These results underscore the role of P8W48 as a nanoreactor, facilitating reversible structural transformations of Pd while preserving its confinement within the cavity.

### 2.3 Selective Hydrogenation

Chemoselective hydrogenation of *α,β*‐unsaturated aldehydes and ketones remains a significant challenge due to the concurrent presence of reducible C═O and C═C functional groups. Although Pd catalysts typically exhibit higher reactivity toward C═C bond hydrogenation, selectively inhibiting C═O hydrogenation is difficult, particularly when the C═C bond bears substituents.^[^ ^25^ ^]^ To evaluate the catalytic performance of Pd8‐H_2_ for hydrogenating α,β‐unsaturated carbonyl compounds, cinnamaldehyde (**1** **a**) was chosen as a model substrate (**Table** **1**). When Pd8‐H_2_ was used as a heterogeneous catalyst in diethyl ether under a 1‐atm H_2_ atmosphere at room temperature (≈25 °C), selective hydrogenation of the C═C bond occurred, yielding hydrocinnamaldehyde (**2** **a**) with 92% yield (Table 1, entry 1). Minimal amounts of C═O‐reduced products, such as hydrocinnamic alcohol (**3** **a**) and cinnamyl alcohol (**4** **a**), were detected. In contrast, no reaction was observed in the absence of a catalyst (Table 1, entry 2), and TBA‐P8W48 alone exhibited no catalytic activity (Table 1, entry 3), confirming that the Pd species within the P8W48 framework serve as the active sites. Previously reported Pd nanoparticles stabilized by a lacunary POM [SiW_9_O_34_]^10−^ (Pd‐SiW9, *d*: ≈ 2.4 nm), synthesized by our group,^[^ ^17c^ ^]^ exhibited negligible activity (Table 1, entry 4), likely due to POM coverage of the Pd surface, which hindered substrate access. A commercial Pd/C catalyst (*d* ≈ 2.7 nm),^[^ ^26^ ^]^ displayed high activity but poor selectivity, yielding **2** **a** in 60% yield and **3** **a** in 31% upon complete conversion of **1** **a** (Table 1, entry 5). Lindlar's catalyst (Pd/CaCO_3_–Pb, without quinoline treatment) showed high selectivity toward **2** **a** (90% selectivity) but significantly lower catalytic activity (17% yield of **2** **a**) (Table 1, entry 6), likely due to surface poisoning and low Pd atom utilization efficiency. These results underscore the critical importance of exposed Pd surfaces in catalytic activity, as passivated Pd particles result in poor performance. In contrast, the superior performance of Pd8‐H_2_ can be attributed to its small cluster size and discrete, accessible surface.

**Table 1:** Hydrogenation of **1** **a** using different catalysts.

Entry	Catalyst	Conversion [%]	Yield [%]		
		1**a**	2**a**	3**a**	4**a**
1	Pd8‐H_2_	>99	92	5	n.d.
2	No catalyst	<1	n.d.	n.d.	n.d.
3	TBA‐P8W48	<1	n.d.	n.d.	n.d.
4	Pd‐SiW9	5	n.d.	n.d.	n.d.
5	Pd/C	>99	60	31	n.d.
6	Lindlar's catalyst	20	17	2	n.d.
7	Pd8	71	66	3	n.d.

(Reaction conditions: **1** **a** (0.5 mmol), catalyst (Pd: 0.5 mol%), diethyl ether (3 mL), biphenyl (0.25 mmol), room temperature (≈25 °C), H_2_ (1 atm), 18 h. Conversions and yields were determined by gas chromatography (GC) using biphenyl as an internal standard (n.d. = not detected).

When Pd8 was used for the reaction under the conditions described in Table 1, the reaction mixture gradually changed from yellow to blackish over 2 h. Although Pd8 exhibited selectivity similar to Pd8‐H_2_, the conversion of 1a was notably lower (Table 1, entry 1 vs entry 7). Reaction profiles indicated a higher initial rate for Pd8‐H_2_, whereas Pd8 exhibited an induction period, likely due to the in situ reduction of Pd^2+^ into Pd^0^ (Figure S8, Supporting Information). Following this induction period, the reaction rates for both catalysts converged, suggesting the formation of the same active Pd nanocluster in situ. Reoxidized Pd8* also catalyzed the reaction, displaying a shorter induction period than Pd8‐H_2_ (Figure S8, Supporting Information), which was consistent with the in situ XANES results indicating a more rapid reduction to Pd^0^ (Figure 6b). After the induction period, the reaction rate and selectivity for **2a** closely aligned with those of Pd8‐H_2_, demonstrating the structural robustness and redox recyclability of Pd8‐H_2_.

**Figure 7:**
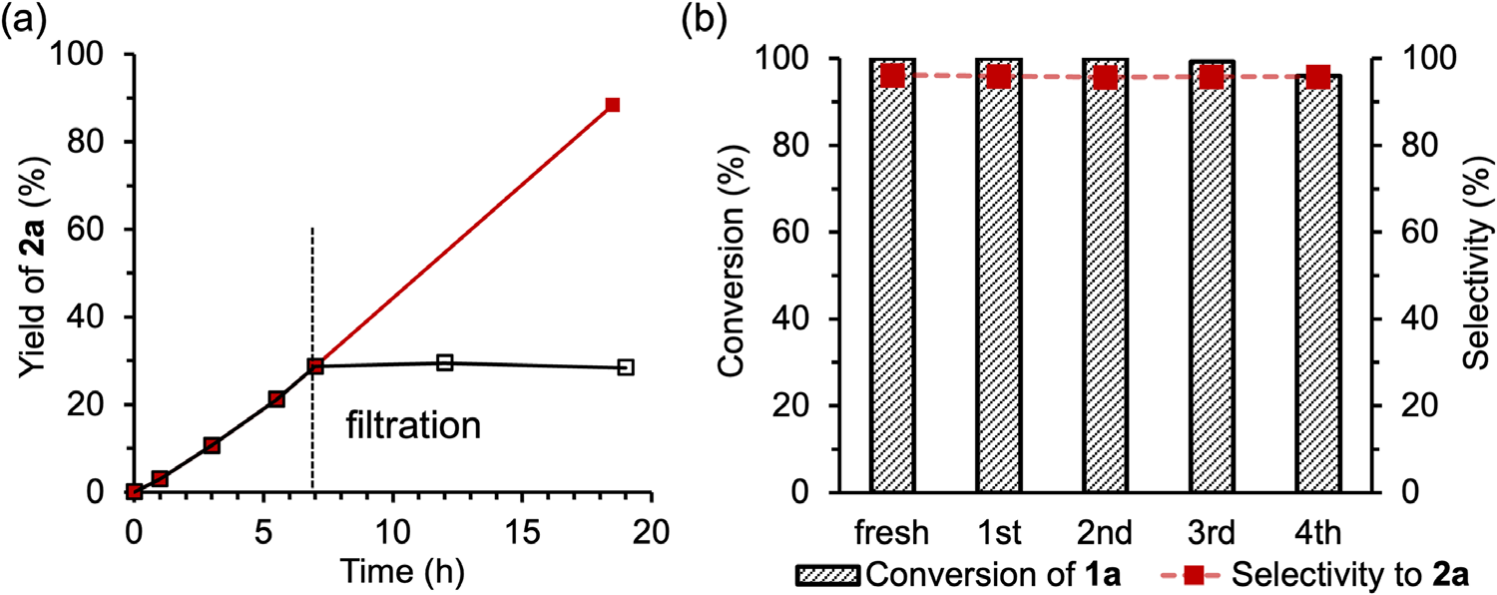
a) Leaching and b) recyclability test of the Pd8‐H_2_ catalyst in hydrogenation of **1** **a**. Reaction conditions: **1** **a** (0.5 mmol), Pd8‐H_2_ (Pd: 0.5 mol%), diethyl ether (3 mL), biphenyl (0.25 mmol), room temperature (≈25 °C), H_2_ (1 atm), 19 h. Conversions and yields were determined by GC using biphenyl as an internal standard.

The heterogeneous nature of Pd8‐H_2_ during hydrogenation of **1** **a** under the conditions outlined in Table 1 was further examined. Filtration of the catalyst at approximately 30% conversion quenched the reaction, with no additional hydrogenation observed under identical conditions (**Figure** **7**a). Inductively coupled plasma‐atomic emission spectroscopy confirmed that no detectable Pd species were present in the filtrate (below the detection limit), further confirming the truly heterogeneous nature of Pd8‐H_2_. The reusability of Pd8‐H_2_ was then assessed. After the reaction, the catalyst was recovered by filtration, washed with diethyl ether, and reused in successive reaction cycles. The recovered Pd8‐H_2_ consistently maintained its catalytic performance for the hydrogenation of **1** **a** over multiple cycles (Figure 7b). Moreover, the selectivity toward **2** **a** remained stable throughout the reaction cycles, indicating the structural integrity of the Pd nanocluster. This stability was additionally supported by time‐profile data, which showed consistent selectivity during the reaction with the reused catalyst (Figure S9, Supporting Information).

**Figure 8:**
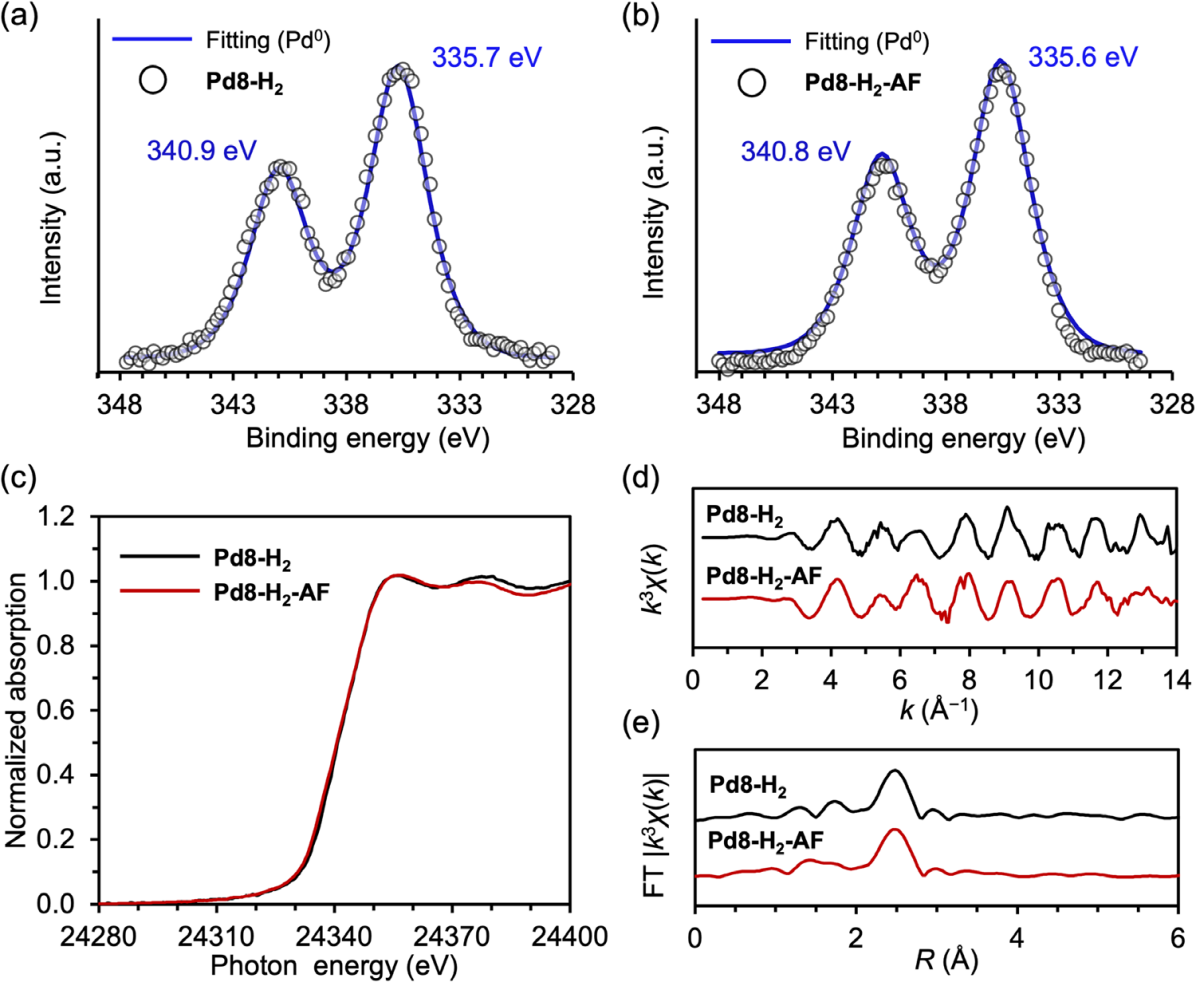
XPS spectra of a) Pd8‐H_2_ and b) recovered Pd8‐H_2_ after the hydrogenation of **1** **a** (Pd8‐H_2_‐AF). c) Pd K‐edge XANES spectra, d) Pd K‐edge *k*‐space, and e) *R*‐space EXAFS spectra of Pd8‐H_2_ and Pd8‐H_2_‐AF.

The integrity of the P8W48 framework after catalysis was confirmed by W L_3_‐edge XAS and IR spectroscopy, which revealed no significant spectral changes compared to fresh Pd8‐H_2_ (Figures S10 and S11, Supporting Information). Further investigation of the structure and electronic state of the Pd nanocluster after catalysis was conducted using XPS, XANES, and EXAFS analyses. The XPS spectrum of the recovered catalyst after hydrogenation of **1** **a** (Pd8‐H_2_‐AF) showed only a minor shift in the Pd 3d_5/2_ binding energy from 335.6 eV (fresh Pd8‐H_2_) to 335.5 eV (Pd8‐H_2_‐AF), indicating minimal change in the electronic state (**Figure** **8**a,b). Similarly, the Pd K‐edge XANES spectra of Pd8‐H_2_‐AF and fresh Pd8‐H_2_ were nearly identical (Figure 8c). Minor changes in the oscillation patterns of the Pd K‐edge *k*‐space EXAFS spectra suggested slight atomic rearrangement (Figure 7d). Additionally, the Pd–Pd CN of 2.2 ± 0.3 in Pd8‐H_2_‐AF confirmed the retention of the small Pd nanocluster within the P8W48 framework (Figure 8e and Table S2, Supporting Information). These results collectively demonstrate the exceptional structural and electronic stability of Pd8‐H_2_ under catalytic conditions.

**Scheme 1:**
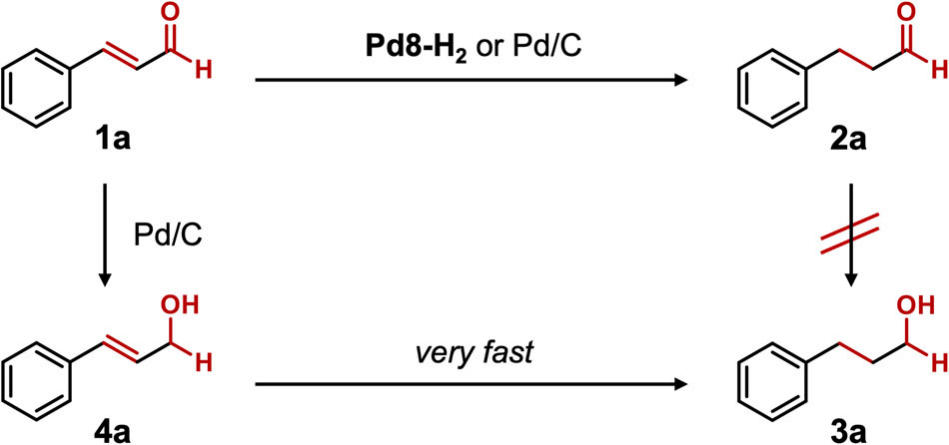
Reaction pathways for the hydrogenation of **1** **a** using Pd8‐H_2_ or Pd/C.

To investigate the origin of the differing reactivity between Pd8‐H_2_ and Pd/C, we examined the hydrogenation of **2** **a**. When **2** **a** was hydrogenated using Pd8‐H_2_, the C═O bond remained intact with less than 1% conversion (Table S3, Supporting Information, entry 1). Similarly, Pd/C produced only a trace amount of **3** **a** (3% yield) by the hydrogenation of **2** **a** (Table S3, Supporting Information, entry 2). These results suggest that **3a** does not form through sequential overhydrogenation of **2** **a**. Indeed, **4** **a** was not detected during the reaction (Figure S8, Supporting Information), and when **4** **a** was subjected to the same conditions, it quickly converted to **3** **a** (Table S4, Supporting Information). These observations indicate that **4** **a** is the intermediate for forming **3** **a** and that the selectivity toward **2** **a** or **3** **a** is determined by the hydrogenation of the C═C or C═O bond of **1** **a**, respectively (**Scheme** **1**).

**Scheme 2:**
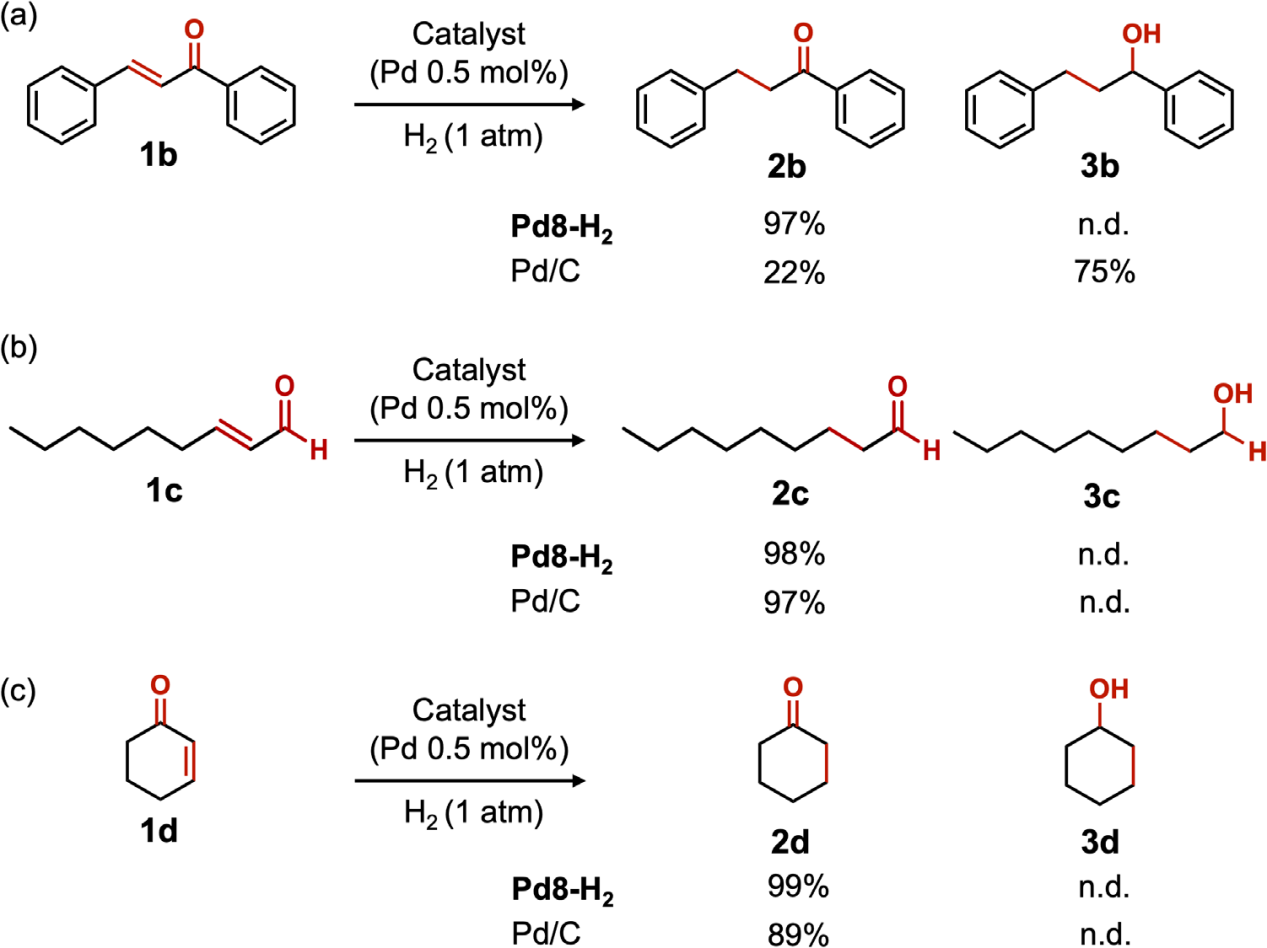
Hydrogenation of a) **1** **b**, b) **1** **c**, and c) **1** **d** using Pd8‐H_2_ and Pd/C. Reaction conditions: substrates (0.5 mmol), catalyst (Pd: 0.5 mol%), diethyl ether (3 mL), room temperature (≈25 °C), H_2_ (1 atm), 9 h. n.d. = not detected.

In the hydrogenation of chalcone (**1** **b**), a representative α,β‐unsaturated ketone, Pd8‐H_2_ exclusively produced dihydrochalcone (**2** **b**) without reducing the C═O bond, whereas Pd/C predominantly yielded over‐hydrogenated product 1,3‐diphenylpropan‐1‐ol (**3** **b**) (**Scheme** **2**a). Notably, even after prolonged reaction times, Pd8‐H_2_ retained its selectivity and did not produce **3** **b**, while Pd/C promoted the overhydrogenation of **2** **b** to **3** **b** (Table S5, Supporting Information). By contrast, in the hydrogenation of 2‐nonenal (**1** **c**) and 2‐cyclohexen‐1‐one (**1** **d**), both Pd8‐H_2_ and Pd/C selectively hydrogenated the C═C bond to afford nonanal (**2c**) and cyclohexanone (**2** **d**), respectively, without overhydrogenation (Scheme 2b,c and Tables S6 and S7, Supporting Information). These results suggest that the presence of a phenyl group influences product selectivity.

The adsorption mode of α,β‐unsaturated aldehydes and ketones on Pd surfaces is crucial for determining hydrogenation selectivity.^[^ ^27^ ^]^ Pd K‐edge XANES spectra indicated that Pd8‐H_2_ and Pd/C share similar electronic states, as evidenced by their overlapping white‐line peaks (≈24356 eV) (Figure S12a, Supporting Information,). Curve‐fitting analysis of EXAFS data revealed identical Pd–Pd distances (2.75 Å) in both catalysts; however, Pd/C exhibited a higher CN (5.1 ± 0.2; Figure S12b,c and Table S2, Supporting Information), consistent with its larger particle size (2.7 nm).^[^ ^26^ ^]^ These structural differences significantly influence the adsorption behavior on the Pd surfaces. Previous studies have shown that the η^4^ adsorption mode (binding both C═C and C═O bonds) is the most stable configuration for cinnamaldehyde on a Pd(111) surface,^[^ ^27^ ^]^ facilitating hydrogenation of both functional groups. The phenyl ring further stabilizes this η^4^ configuration through π interactions with the extended flat Pd surface, leading to the abovementioned poor selectivity in the hydrogenation of **1** **a** and **1** **b** by Pd/C (Figure S13a, Supporting Information). In contrast, the discrete surface structure of Pd8‐H_2_ suppresses η^4^ adsorption and favors η^2^ adsorption of the C═C bond, resulting in selective hydrogenation of the C═C bond (Figure S13b, Supporting Information). Density function theory studies have shown that phenyl ring adsorption is suppressed on ultrasmall Pd nanoclusters.^[^ ^28^ ^]^ For **1** **c**, the flexible alkyl chain destabilizes η^4^ adsorption, making η^2^ adsorption favorable even on extended Pd surfaces.^[^ ^27^ ^]^ As a result, both Pd/C and Pd8‐H_2_ exhibit similar selectivity for the saturated aldehyde **2** **c** in the hydrogenation of **1** **c** (Figure S13c,d, Supporting Information). These findings underscore the effects of differences in Pd surface structures on adsorption behavior and hydrogenation selectivity in substrates containing aromatic groups.

**Figure 9:**
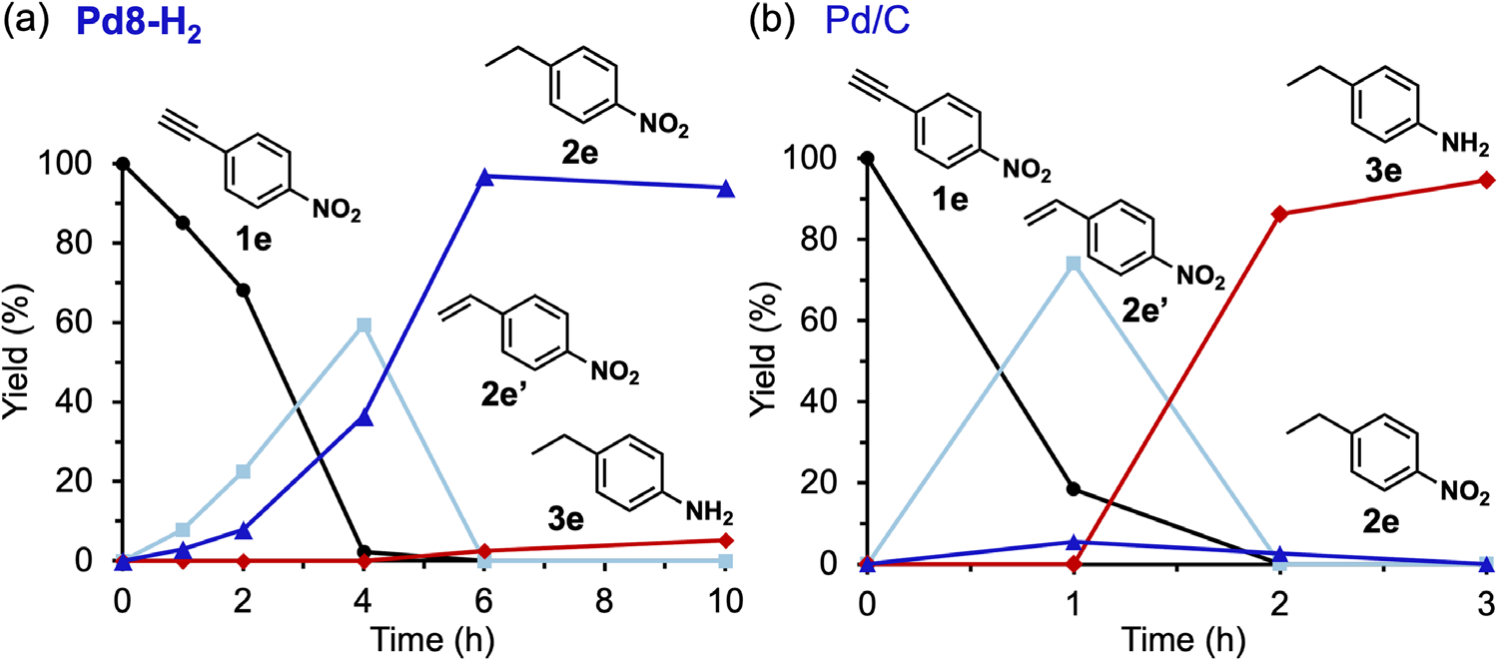
Time profiles of the hydrogenation of **1** **e** catalyzed by a) Pd8 and b) Pd/C. Reaction conditions: **1** **e** (0.5 mmol), catalyst (Pd: 0.5 mol%), diethyl ether (3 mL), biphenyl (0.25 mmol), room temperature (≈25 °C), H_2_ (1 atm).

The hydrogenation of 1‐ethynyl‐4‐nitrobenzene (**1** **e**) was investigated further to evaluate the chemoselectivity of Pd8‐H_2_. Under 1‐atm H_2_ in diethyl ether, Pd8‐H_2_ selectively hydrogenated the C≡C bond of **1** **e** to yield 4‐nitrostyrene (**2** **e’**), which was further reduced to 4‐ethylnitrobenzene (**2** **e**) (**Figure** **9**a). Notably, over‐hydrogenated products resulting from nitro group hydrogenation, such as 4‐ethylaniline (**3** **e**), were scarcely detected even after complete conversion of **1** **e**. In contrast, when Pd/C was used under identical conditions, the nitro group was rapidly hydrogenated, leading to the selective formation of **3** **e** after **2** **e’** and **2** **e** intermediate formation (Figure 9b). Similar to the selective hydrogenation of **1** **a**, this difference in selectivity may again be attributed to the phenyl group of **2** **e**. After full conversion of **1**e into **2** **e**, the planar interaction between the phenyl ring and the extended Pd surface of Pd/C facilitates the adsorption of **2** **e**, promoting its further hydrogenation to form **3** **e** (Figure S13e, Supporting Information). In contrast, the discrete surface of Pd8‐H_2_ interacts weakly with the phenyl ring, enabling rapid desorption of **2** **e** from active sites and preventing overhydrogenation (Figure S13f, Supporting Information). These findings highlight the unique surface characteristics of Pd8‐H_2_, which favor selective adsorption and hydrogenation of C≡C and C═C bonds, accounting for its distinctive catalytic behavior.

## 3 Conclusion 

In this study, we developed a strategy to fabricate a surface‐exposed, small Pd nanocluster confined within the cavity of a ring‐shaped POM [P_8_W_48_O_184_]^40−^. The synthetic approach involved preparing a Pd^2+^ precursor (Pd8), followed by solid‐state reduction using H_2_ gas under mild conditions (1 atm H_2_, ≈25 °C) to form the Pd nanocluster Pd8‐H_2_. Comprehensive characterization using SCXRD, XPS, XAS, CO‐DRIFTS, atomic‐resolution STEM, and UV–vis confirmed that Pd8‐H_2_ consists of a small Pd nanocluster with an exposed, discrete metal surface. This unique surface structure enabled Pd8‐H_2_ to function as a highly selective heterogeneous catalyst. It demonstrated exceptional chemoselectivity in the hydrogenation of cinnamaldehyde to hydrocinnamaldehyde and 1‐ethynyl‐4‐nitrobenzene to 4‐ethylnitrobenzene, outperforming commercial Pd/C catalysts. Recyclability studies and post‐reaction analyses demonstrated the high structural and electronic stability of the Pd nanocluster during catalysis. The rigid POM molecular templates not only provided a confined nanospace for cluster formation but also imparted enhanced thermal and chemical robustness of the resulting catalyst. This strategy offers a promising approach for designing advanced metal nanocluster catalysts, providing precise surface control and superior selectivity for diverse molecular transformations.

## Conflict of Interest

The authors declare no conflict of interest.

## Supporting information



Supporting Information

## Data Availability

The data that support the findings of this study are available in the Supporting Information of this article.
