# Supplementary material for: Surface‐Exposed Pd Nanocluster Confined within a Ring‐Shaped Polyoxometalate for Selective Hydrogenation
Source: Adv Sci (Weinh). 2025 Jul 28;12(38):e09418. doi: 10.1002/advs.202509418 (PMC12520513; doi:10.1002/advs.202509418)
Supplement: Supplementary file 1 — Supporting Information [file ADVS-12-e09418-s001.pdf]

ADVANCED SCIENCE
Open Access

# Supporting Information

for *Adv. Sci.*, DOI 10.1002/advs.202509418

Surface-Exposed Pd Nanocluster Confined within a Ring-Shaped Polyoxometalate for Selective Hydrogenation

*Rui Xi, Kentaro Yonesato, Takafumi Yatabe, Yoshihiro Koizumi, Soichi Kikkawa, Seiji Yamazoe, Koji Harano, Kazuya Yamaguchi and Kosuke Suzuki**

*Supporting Information for*

# Surface-Exposed Pd Nanocluster Confined within a Ring-Shaped Polyoxometalate for Selective Hydrogenation

Rui Xi,[1] Kentaro Yonesato,[1,5] Takafumi Yatabe,[1] Yoshihiro Koizumi,[1] Soichi Kikkawa,[2] Seiji Yamazoe,[2] Koji Harano,[3,4] Kazuya Yamaguchi,[1] Kosuke Suzuki*[1,5]

[1] Department of Applied Chemistry, School of Engineering, The University of Tokyo
7-3-1 Hongo, Bunkyo-ku, Tokyo 113-8656 (Japan)

[2] Department of Chemistry, Graduate School of Science, Tokyo Metropolitan University
1-1 Minami Osawa, Hachioji, Tokyo 192-0397 (Japan)

[3] Center for Basic Research on Materials, National Institute for Materials Science (NIMS)
1-1 Namiki, Tsukuba, Ibaraki 305-0044 (Japan)

[4] Research Center for Autonomous Systems Materialogy (ASMat), Institute of Integrated Research
Institute of Science Tokyo
4259 Nagatsuda-cho, Midori-ku, Yokohama, Kanagawa 226-8501 (Japan)

[5] Department of Advanced Materials Science, Graduate School of Frontier Science, The University of Tokyo
5-1-5 Kashiwanoha, Kashiwa, Chiba 277-8561 (Japan)

* E-mail: ksuzuki@appchem.t.u-tokyo.ac.jp

| Contents | Page |
|---|---|
| Experimental section | S2–S5 |
| Tables S1–S7 | S6–S9 |
| Figures S1–S13 | S10–S16 |
| References | S17 |

## Experimental section

### Materials

Tetra-*n*-butylammonium (TBA) hydroxide 30-hydrate (TBAOH·30$H_2O$), palladium acetate and palladium (5 wt. % on calcium carbonate, poisoned with lead (Pd/$CaCO_3$–Pb)) were purchased from Merck Sigma-Aldrich. Acetone, acetonitrile, *N,N*-dimethylformamide (DMF), and diethyl ether were purchased from Kanto Chemical Co. Inc.. All solvents were dried and stored with appropriate molecular sieves. Cinnamaldehyde, hydrocinnamaldehyde, cinnamyl alcohol, 2-nonenal, chalcone, 2-cyclohexen-1-one, biphenyl, phenylacetylene, styrene, ethylbenzene, nitrobenzene, and chlorobenzene were purchased from Tokyo Chemical Industry Co. Ltd.. TBA salt of ring-shaped $[P_8W_{48}O_{184}]^{40-}$ (**TBA-P8W48,** $TBA_{12}H_{28}[P_8W_{48}O_{184}]$), was synthesized according to the reported procedures.[S1]

### Instruments

The elemental analysis of P, W, and Pd was performed on a Shimadzu ICP-8100 instrument using Argon gas as the plasma gas and carrier gas. The Fourier-transform infrared (FT-IR) spectra of **Pd8**, **Pd8-H$_2$** and **Pd8*** were obtained using a JASCO FT/IR-4100 instrument with an attenuated total reflectance (ATR) method. CO-adsorption diffuse reflective infrared Fourier-transform spectroscopy (CO-DRIFTS) of **Pd8-H$_2$** was carried out a JASCO FT/IR-6700 instrument by the introduction of CO gas with a pressure around 15 psi to the sample at room temperature. The diffusion reflectance ultraviolet-visible (UV-vis) spectra were obtained by using a JASCO V-770 instrument and using barium sulfate as a background. In the *in-situ* experiment was carried out under 10 mL/min $H_2$ gas flow. X-ray photoelectron spectroscopy (XPS) measurements were performed using a JEOL JPS9030 instrument. The binding energies were corrected using C 1s region (284.8 eV). The curve fitting analysis of XPS spectra were performed with a Gauss–Lorentz function after removing the background using Shirley method. Gas Chromatography (GC) analyses were obtained using a Shimadzu GC-2014 instrument equipped with a flame ionization detector (FID) and a capillary column (GL Science, InertCap5; 0.25 mm x 30 m). Shimadzu Chromatopack C-R8A was used to calculate the peak area. GC-mass performed using a Shimadzu GCMS-QP2020 equipped with a GL Science InertCap5 capillary column (0.25 mm x 30 m) at an ionization voltage of 70 eV.

### X-ray crystallographic analysis

The single-crystal X-ray diffraction experiments of **Pd8** and **Pd8-H$_2$** were conducted at the BL02B1 beamline of SPring-8 facility of the Japan Synchrotron Radiation Research Institution (Proposal number: 2023B1842, 2024A1880, 2024B1868). The incident X-ray was monochromatized using a silicon (311) double-crystal monochromator (λ = 0.4134 Å for **Pd8**, 0.4124 Å for **Pd8-H$_2$**). The diffraction experiments were carried out using a DECTRIS PILATUS3 X CdTe 1M detector with the RAPID program at 100K. The SC-XRD experiment of **Pd8*** was carried out on a Rigaku XtaLab Synergy-R diffractometer equipped with a rotating anode Mo K$\alpha$ irradiation (λ = 0.71073 Å, 50 kV, 24 mA) with CrysAlisPro Program.[S2] Data processing was carried out using CrysAlisPro program,[S2] including empirical absorption correction and Lorentz correction. Structural analysis was performed using Olex$^2$ program[S3] and WinGX program.[S4] All structures were solved by SHELXT-2018/2 (intrinsic phase methods)[S5] and refined by SHELXL-2018/3.[S6] All atoms were refined as anisotropic. The highly disordered TBA cations and solvent molecules were omitted by using SQUEEZE program.[S7] CCDC-2449692 contains the supplementary crystallographic data for **Pd8**, which can be

obtained free of charge from The Cambridge Crystallographic Data Centre via www.ccdc.cam.ac.uk/data_request/cif. Since the molecular structures of **Pd8-H₂** and **Pd8*** were not completely determined due to the severe disorder of possible Pd sites within a cavity of **P8W48**, these data are not deposited in CCDC but provided as supporting materials.

**Bond valence sum (BVS) calculation**

The BVS values were calculated by the expression for the variation of the length $r_{ij}$ of a bond between two atoms *i* and *j* in observed crystal with valence $V_i$ using equation:

$$V_i = \sum_j \exp\left(\frac{r'_0 - r_{ij}}{B}\right)$$

where *B* is a constant equal to 0.37 Å, $r'_0$ is bond valence parameter for a given atom pair.[S8]

**X-ray absorption fine structure (XAFS)**

Pd K-edge and W $L_3$-edge XAFS were performed at the BL01B1 beamline of SPring-8 facility of the Japan Synchrotron Radiation Research Institution (Proposal number: 2022B1860, 2023A1732, 2023B1651, 2024B1879, 2023B2070, 2024A1775, 2024B1775). The incident X-ray beam was monochromatized by using Si(111) and Si(311) double-crystal monochromator for Pd K-edge and W $L_3$-edge, respectively. X-ray absorption near-edge structure (XANES) and extended XAFS (EXAFS) spectra were analyzed using the xTunes program,[S9] Pre-edge background and EXAFS background were subtracted using McMaster and Cubicspline methods, respectively. The XANES spectra were obtained after normalization at edge height. The *k*-space EXAFS spectra were obtained as $k^3$-weighted spectra. The *R*-space EXAFS spectra were obtained by the Fourier-transformation in the k range of 3–14 $Å^{-1}$ and 3–16 $Å^{-1}$ for Pd K-edge and W $L_3$-edge, respectively. Curve fitting analysis for Fourier transformed EXAFS spectra were carried out for back-*k*-space oscillations conducted using the FEFF8 program (details of the curve-fitting analysis are provided in Figure 3e, Table S2).[S10] $R_f = \{\Sigma[k^3\, \chi_{obs}(k) - k^3\, \chi_{cal}(k)]^2 / \Sigma[k^3\, \chi_{obs}\, (k)]^2\}^{1/2}$ where, $\chi_{obs}$ and $\chi_{cal}$ correspond to the observed and calculated data, respectively. In the *in situ* experiment, the sample was first heated to 50 °C under 30 mL/min helium (He) gas flow, followed by switching gas to $H_2$ at the same flow rate. In changing the gas flow between $H_2$ and $O_2$, the flow path was once exchanged to He to prevent hydrogen explosion.

**Scanning transmission electron microscopy (STEM) and energy dispersive X-ray spectroscopy (EDS)**

Annular dark field scanning transmission electron microscopic (ADF-STEM) imaging was carried out by using an aberration-corrected transmission electron microscope (Themis Z, Thermo Fisher Scientific, Inc.) at an acceleration voltage of 300 kV, a convergence semi-angle to 17.9 mrad, a collection semi-angle to 31-190 mrad, and a probe current to 4-7 pA. The STEM images were processed by using a Gaussian blur filter for denoising. STEM-EDS analysis was carried out by using an aberration-corrected transmission electron microscope (Spectra Ultra, Thermo Fisher Scientific, Inc.) equipped with an Ultra-X EDS detector at an acceleration voltage of 60 kV, a convergence semi-angle to 30.0 mrad, and a probe current to 3.3 nA. Data analysis of EDS spectra was carried out on Velox software (Thermo Fisher Scientific, Inc.) using a single three-parameter Bethe-Heitler function as a background correction parameter.

The specimen for the STEM experiments were prepared by drop-casting acetonitrile dispersion of

**Pd8-H$_2$** onto a TEM microgrid covered with an amorphous carbon film (SHR-C075, Okenshoji Co. Ltd.).

**Synthesis of TBA$_{16}$H$_8$[Pd$_8$P$_8$W$_{48}$O$_{184}$]·12H$_2$O·6C$_3$H$_6$O (Pd8)**

**TBA-P8W48** (TBA$_{12}$H$_{28}$[P$_8$W$_{48}$O$_{184}$]; 116.1 mg, 7.5 μmol) and TBAOH·30H$_2$O (40.5 mg, 45.0 μmol) were dissolved in acetone (3.0 mL). The resulting solution was stirred for 10 min at room temperature (~25 °C), followed by addition of palladium acetate (26.9 mg, 120 μmol) and stirring for further 3 days. Diethyl ether (~3 mL) was added to the resulting solution after the filtration and allowed stand for 1 day. Block-shaped orange crystalline product (**Pd8**) was obtained (43.1 mg, 37% yield). IR (ATR): 3433, 2963, 2935, 2872, 1698, 1634, 1552, 1485, 1467, 1378, 1140, 1071, 1028, 977, 882, 832, 718, 695, 538 cm$^{-1}$. Elemental analysis: calcd (%) for TBA$_{16}$H$_8$[Pd$_8$P$_8$W$_{48}$O$_{184}$]·12H$_2$O·6C$_3$H$_6$O: C, 19.00; H, 3.75; N, 1.29; P, 1.43; W, 50.96; Pd, 4.89. Found: C, 18.83; H, 3.70; N, 1.27; P, 1.41; W, 50.91; Pd, 4.95.

**Preparation of hydrogen-reduced Pd8 (Pd8-H$_2$)**

Solid sample of **Pd8** was kept in a round-bottom flask at room temperature (~25 °C) for 30 min under H$_2$ (1 atm). The black product (**Pd8-H$_2$**) was collected under Ar. For the crystallographic and elemental analysis, **Pd8-H$_2$** was recrystallized in a mixed solvent of acetone and diethyl ether under an Ar atmosphere (21% yield). IR (ATR) for **Pd8-H$_2$**: 3433, 2963, 2935, 2872, 1698, 1634, 1552, 1485, 1467, 1378, 1140, 1071, 1028, 977, 882, 832, 718, 695, 538, 467 cm$^{-1}$. Elemental analysis calcd (%) for TBA$_{16}$H$_{24}$[Pd$_8$P$_8$W$_{48}$O$_{184}$]·12H$_2$O·6C$_3$H$_6$O: P, 1.42; W, 50.91; Pd, 4.89. Found: P, 1.39; W, 53.00; Pd, 4.77.

**Preparation of the reoxidized sample of Pd8-H$_2$ (Pd8*)**

Solid sample of **Pd8-H$_2$** was kept in a round-bottom flask at room temperature (~25 °C) for 1 day under air (1 atm). The brown product (**Pd8***) was collected. Single-crystal X-ray diffraction experiment of **Pd8*** was carried out on the single crystal of **Pd8-H$_2$** after exposure to air for 1 day at room temperature.

**Reduction of Pd8 in acetone solution under H$_2$**

A Schlenk tube, which contained **Pd8** (7.5 μmol) and acetone (3 mL), was degassed and filled with H$_2$ (1 atm). The solution was stirred for 15 min at room temperature. Then, by addition of diethyl ether to the solution, single crystals were obtained. Elemental analysis for the crystalline product: found, P 1.43, W 52.87, Pd 1.43 (molar ratio of Pd/**P8W48** = 2/1).

**Typical procedures for catalytic reactions**

Catalyst (Pd: 0.5 mol%), substrate (0.50 mmol), biphenyl (0.25 mmol, internal standard), diethyl ether (3.0 mL), and a Teflon-coated magnetic stirring bar were placed into a Schlenk tube. The reaction mixture was degassed, and the Schlenk tube was filled with H$_2$ (1 atm). The reaction mixture was stirred at ~25 °C. The substrate conversion and the product yield were determined by GC analysis using biphenyl as an internal standard. Sampling was performed using a syringe at predetermined time intervals.

**Leaching test of Pd8-$H_2$ catalyst in hydrogenation**

The hydrogenation reaction was performed by using cinnamaldehyde (**1a**) as a substrate under the same conditions as mentioned above. When the conversion reached approximately 30%, the atmosphere was changed to Ar, and the reaction mixture was divided into equal two parts. For one part of the reaction mixture, the reaction was continued under $H_2$ (1 atm). For the other part, the catalyst was removed by filtration and the filtrate was transferred to a new Schlenk tube. After exchanging the atmosphere to $H_2$ (1 atm), and the reaction was restarted. The conversion of **1a** and the yield of product were determined by GC analysis using biphenyl as an internal standard.

**Recyclability test of Pd8-$H_2$ catalyst in hydrogenation**

The catalyst after the hydrogenation reaction of **1a** was retrieved from the reaction mixture by filtration, followed by washing with diethyl ether and drying by vacuum pumping. All the operations were conducted under an Ar atmosphere. Then, the retrieved catalyst was used for the hydrogenation of **1a** under the same reaction conditions as that using the fresh catalyst.

**Table S1.** Crystallographic parameters of **Pd8**, **Pd8-H$_2$**, and **Pd8***.

| | **Pd8** | **Pd8-H$_2$** | **Pd8*** |
|---|---|---|---|
| Crystal system | Tetragonal | Tetragonal | Tetragonal |
| Space group | *I*4/*m* (#87) | *I*4/*m* (#87) | *I*4/*m* (#87) |
| *a* (Å) | 28.4662(6) | 28.6657(4) | 28.3825(5) |
| *b* (Å) | 28.4662(6) | 28.6657(4) | 28.3825(5) |
| *c* (Å) | 32.8234(18) | 32.5129(7) | 32.1079(13) |
| $\alpha$ (deg) | 90 | 90 | 90 |
| $\beta$ (deg) | 90 | 90 | 90 |
| $\gamma$ (deg) | 90 | 90 | 90 |
| Volume (Å$^3$) | 26597.6(18) | 26716.6(9) | 25865.0(14) |
| *Z* | 2 | 2 | 2 |
| $\rho_{calc}$ (g cm$^{-3}$) | 1.607 | 1.599 | 1.864 |
| Temp (K) | 100(2) | 100(2) | 93(2) |
| GOF | 1.065 | 0.980 | 0.968 |
| $R_1$ [$I > 2\sigma(I)$] [a] | 0.0912 | 0.0437 | 0.0568 |
| $wR_2$[a] | 0.3539 | 0.1562 | 0.2039 |

[a]$R_1 = \Sigma||F_o|-|F_c||/\Sigma|F_o|$, $wR_2 = \{\Sigma[w(F_o^2-F_c^2)]/\Sigma[w(F_o^2)^2]\}^{1/2}$

**Table S2.** Fitting parameters in Pd K-edge EXAFS of **Pd8-H$_2$**, **Pd8-H$_2$** after the hydrogenation of **1a** (**Pd8-H$_2$-AF**), Pd/C, and Pd foil.

| Sample | Path | CN[a] | *R* (Å) | $\Delta E$ (eV) | (DW)$^2$ (10$^{-1}$Å$^2$) | $R_f$ (%) |
|---|---|---|---|---|---|---|
| **Pd8-H$_2$** | Pd···Pd | 1.8(2) | 2.75(4) | –7.3(7) | 0.09(3) | 5.7 |
| **Pd8-H$_2$-AF** | Pd···Pd | 2.2(3) | 2.74(4) | –7.8(6) | 0.09(3) | 5.0 |
| Pd/C | Pd···Pd | 5.1(2) | 2.75(2) | –9.8(4) | 0.08(1) | 8.1 |
| Pd foil | Pd···Pd | 10.6(2) | 2.73(1) | –7.1(2) | 0.07(1) | 9.4 |

*R* range = 2.10–2.86 Å and back *k* range = 3–14 Å$^{-1}$. For Pd foil, *R* range = 1.90–2.85 Å. [a]Coordination number.

**Table S3.** Hydrogenation of hydrocinnamaldehyde (**2a**) by using **Pd8-$H_2$** and Pd/C.

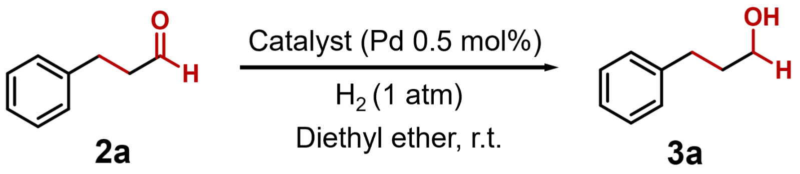


| Entry | Catalyst | Conversion (%) | Yield (%) |
|---|---|---|---|
| | | **2a** | **3a** |
| 1 | **Pd8-$H_2$** | <1 | n.d. |
| 2 | Pd/C | 4 | 3 |

Reaction conditions: **2a** (0.5 mmol), catalyst (Pd: 0.5 mol%), diethyl ether (3 mL), biphenyl (0.25 mmol), room temperature (~25 °C), $H_2$ (1 atm), 18 h (n.d. = not detected).

**Table S4.** Hydrogenation of cinnamyl alcohol (**4a**) by using **Pd8-$H_2$** and Pd/C.

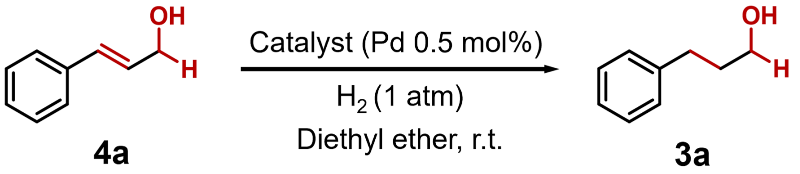


| Entry | Catalyst | Conversion (%) | Yield (%) |
|---|---|---|---|
| | | **4a** | **3a** |
| 1 | **Pd8-$H_2$** | >99 | >99 |
| 2 | Pd/C | >99 | >99 |

Reaction conditions: **4a** (0.5 mmol), catalyst (Pd: 0.5 mol%), diethyl ether (3 mL), biphenyl (0.25 mmol), room temperature (~25 °C), $H_2$ (1 atm), 3 h (n.d. = not detected).

**Table S5.** Hydrogenation of chalcone (**1b**) by using **Pd8-H₂** and Pd/C.

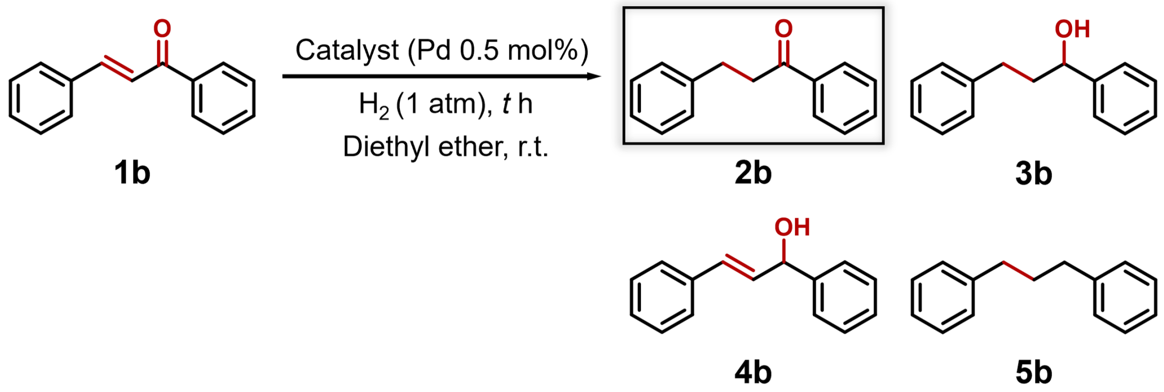


| Entry | Catalyst | Time (h) | Conversion (%) | Yield (%) | | | |
|---|---|---|---|---|---|---|---|
| | | | **1b** | **2b** | **3b** | **4b** | **5b** |
| 1 | **Pd8-$H_2$** | 9 | >99 | 97 | n.d. | n.d. | n.d. |
| 2 | **Pd8-$H_2$** | 18 | >99 | 96 | n.d. | n.d. | n.d. |
| 3 | Pd/C | 9 | >99 | 22 | 75 | n.d. | n.d. |
| 4 | Pd/C | 18 | >99 | n.d. | 87 | n.d. | 6 |

Reaction conditions: **1b** (0.5 mmol), catalyst (Pd: 0.5 mol%), diethyl ether (3 mL), biphenyl (0.25 mmol), room temperature (~25 °C), $H_2$ (1 atm). Conversions and yields were determined by GC using biphenyl as an internal standard (n.d. = not detected).

**Table S6.** Hydrogenation of 2-nonenal (**1c**) by using **Pd8-$H_2$** and Pd/C.

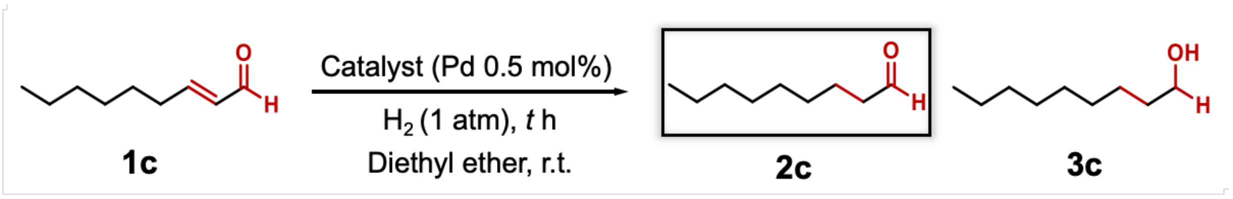


| Entry | Catalyst | Time (h) | Conversion (%) | Yield (%) | |
|---|---|---|---|---|---|
| | | | **1c** | **2c** | **3c** |
| 1 | **Pd8-$H_2$** | 9 | >99 | 98 | n.d. |
| 2 | **Pd8-$H_2$** | 9 | >99 | 97 | n.d. |
| 3 | Pd/C | 18 | >99 | 97 | n.d. |
| 4 | Pd/C | 18 | >99 | 96 | n.d. |

Reaction conditions: **1c** (0.5 mmol), catalyst (Pd: 0.5 mol%), diethyl ether (3 mL), biphenyl (0.25 mmol), room temperature (~25 °C), $H_2$ (1 atm). Conversions and yields were determined by GC using biphenyl as an internal standard (n.d. = not detected).

**Table S7.** Hydrogenation of 2-nonenal (**1d**) by using **Pd8-$H_2$** and Pd/C.

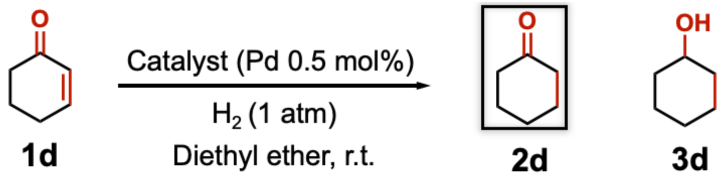


| Entry | Catalyst | Conversion (%) | Yield (%) | |
|---|---|---|---|---|
| | | **1d** | **2d** | **3d** |
| 1 | **Pd8-$H_2$** | >99 | 99 | n.d. |
| 2 | Pd/C | >99 | 89 | n.d. |

Reaction conditions: **1d** (0.5 mmol), catalyst (Pd: 0.5 mol%), diethyl ether (3 mL), biphenyl (0.25 mmol), room temperature (~25 °C), $H_2$ (1 atm), 9 h. Conversions and yields were determined by GC using biphenyl as an internal standard (n.d. = not detected).

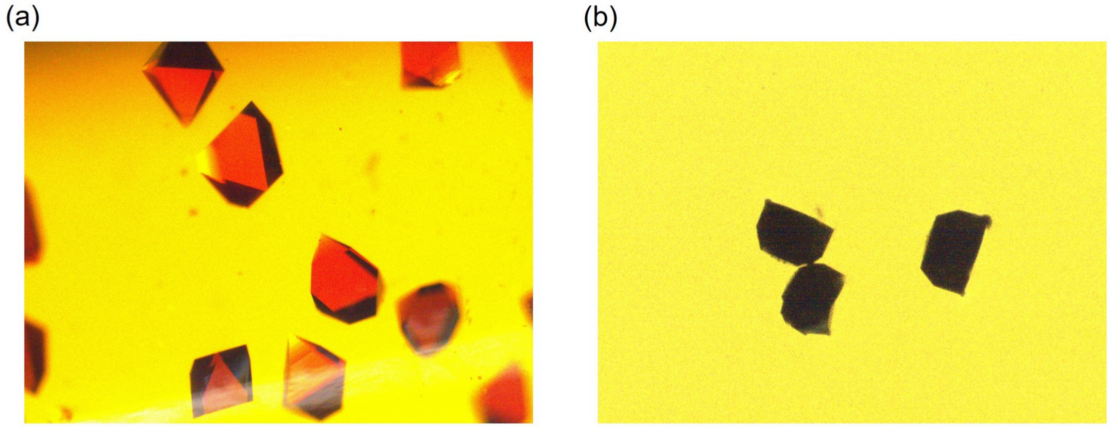


**Figure S1.** Photographs of crystalline (a) **Pd8** and (b) **Pd8-H$_2$**.

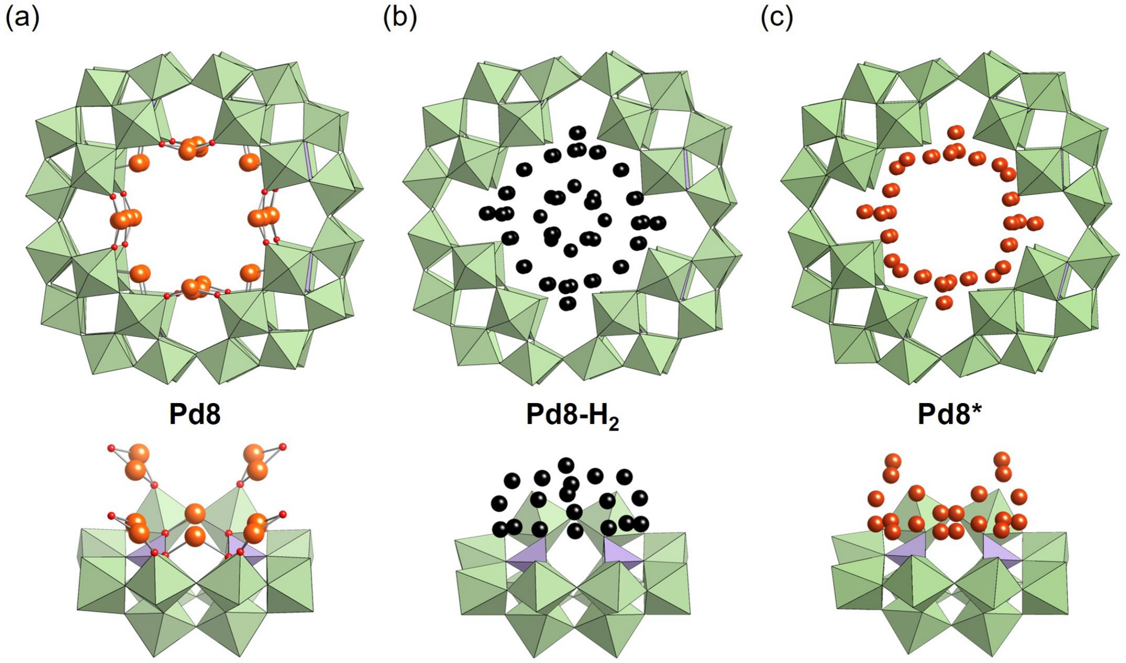


**Figure S2.** (a) Crystal structure of anionic part of **Pd8**. (b,c) Schematic of possible Pd sites in (b) **Pd8-H$_2$** and (c) **Pd8*** based on the crystallographic analysis. Color code: green octahedra, {$WO_6$}; purplue tetrahedra, {$PO_4$}, red sphere O; orange sphere Pd atom; black and maroon sphere possible Pd sites in **Pd8-H$_2$** and **Pd8**.

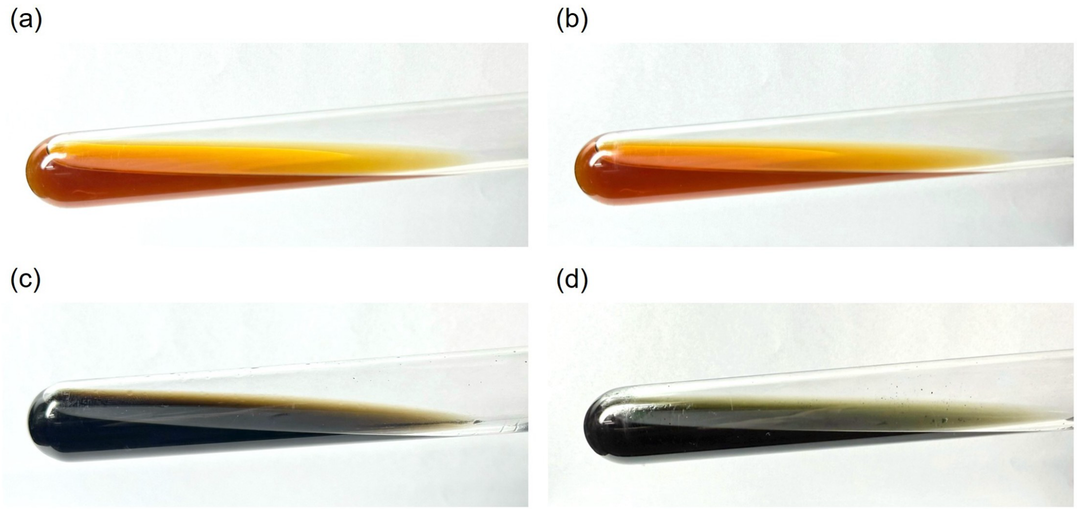


**Figure S3.** Photographs of acetone solutions of (a) **Pd8** and (b) **Pd8** after treating with $H_2$ for 5 min; acetonitrile solutions of (c) **Pd8** and (d) **Pd8** after adding $TBABH_4$.

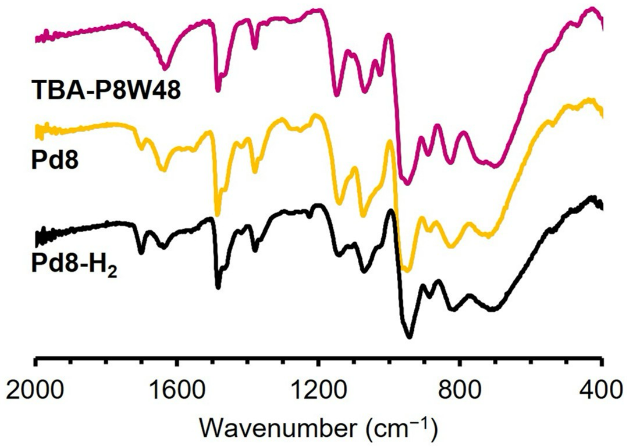


**Figure S4.** IR (ATR method) spectra of **TBA-P8W48** (pink line), **Pd8** (yellow line), **Pd8-$H_2$** (black line).

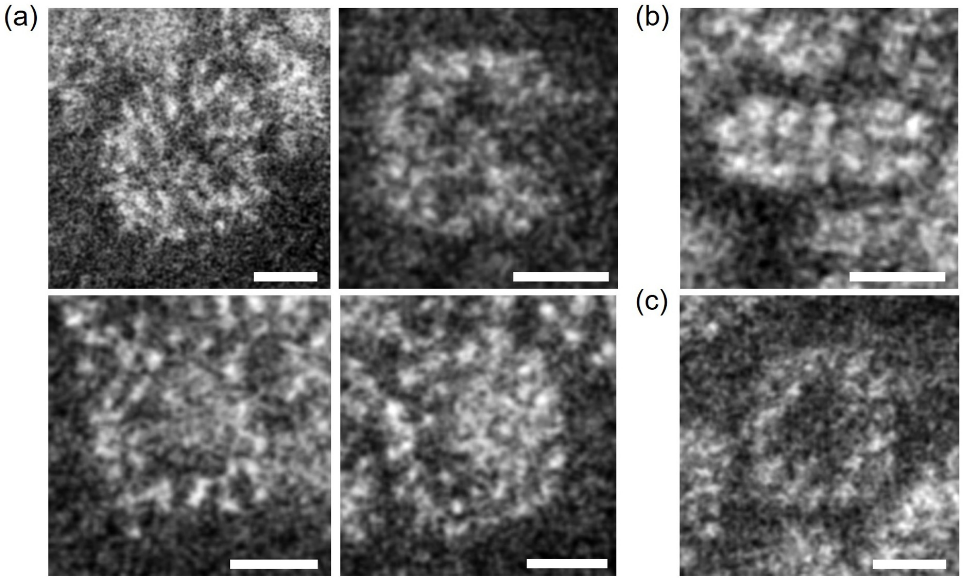


**Figure S5.** ADF-STEM images Pd-incorporated and empty **P8W48**. (a) Individual particles of **Pd8-$H_2$**. (b) A side-on view of **P8W48**. (c) **P8W48** without a Pd cluster. Scale bars: 1 nm.

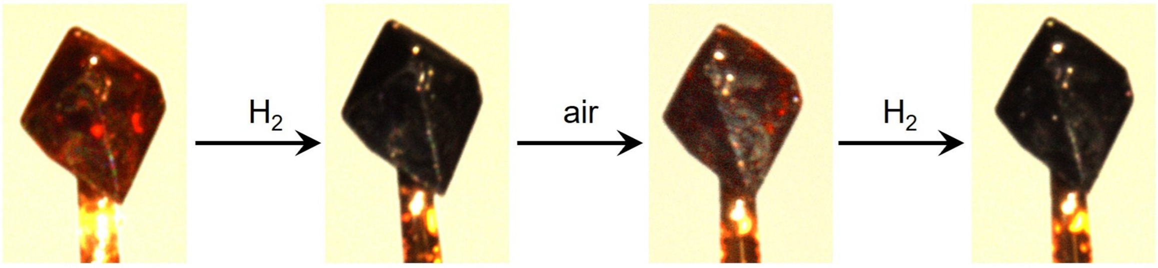


**Figure S6.** Photographs of a single crystal of **Pd8** before and after the treatment by switching the atmosphere with $H_2$ and air at room temperature (~25 °C).

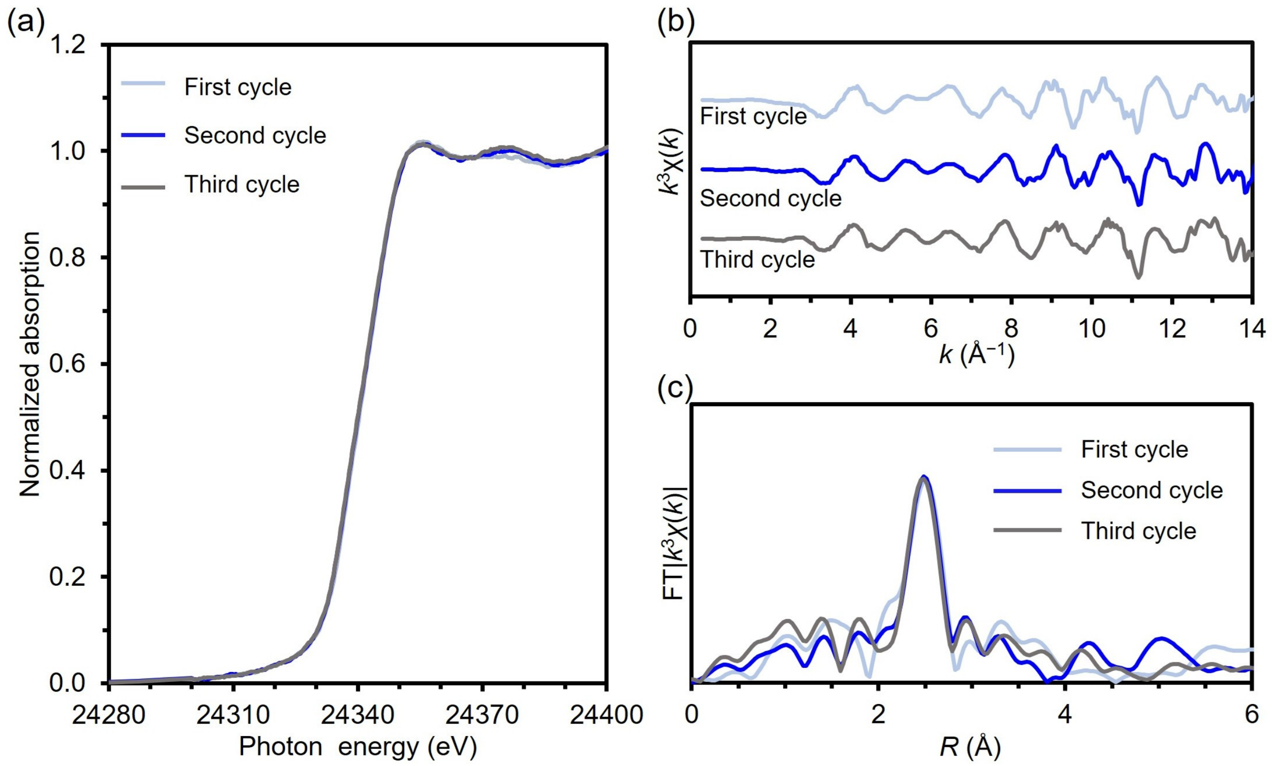


**Figure S7.** (a) Pd K-edge XANES spectra, (b) $k^3$-weighted *k*-space and (c) *R*-space EXAFS spectra of after the hydrogen reduction in i*n-situ* Pd K-edge XAFS study of **Pd8** with switching $H_2$ and $O_2$ gas flows at 50 °C. First cycle (light blue line), second cycle (blue line) and third cycle (black line).

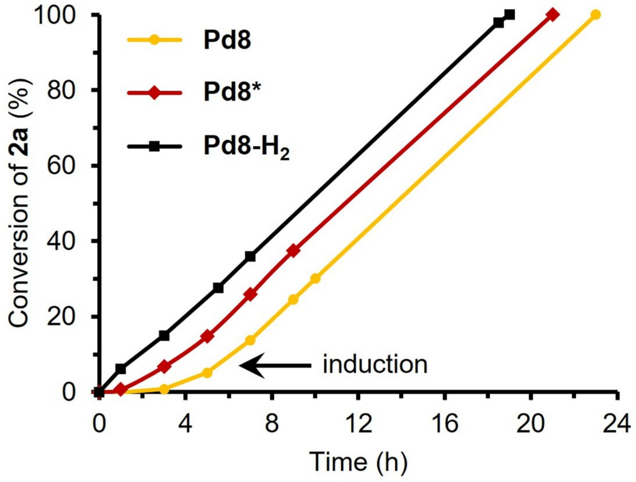


**Figure S8.** Reaction profiles for the hydrogenation of **1a** by **Pd8, Pd8*,** and **Pd8-H₂**. Reaction conditions: **1a** (0.5 mmol), biphenyl (0.25 mmol), catalysts (Pd: 0.5 mol%), diethyl ether (3 mL), room temperature (~25 °C), $H_2$ (1 atm).

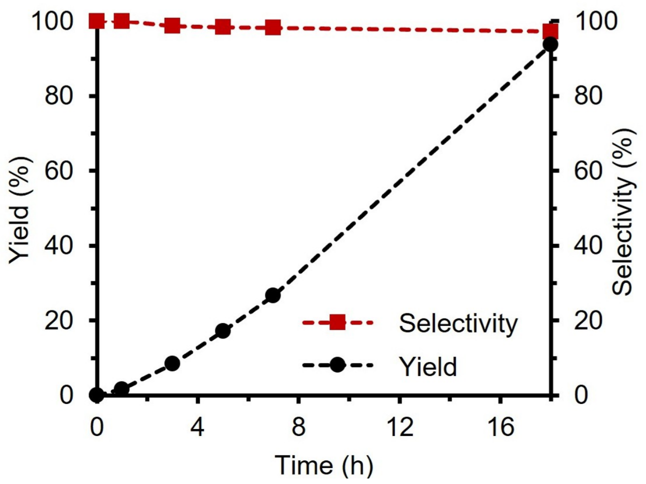


**Figure S9.** Time profile for the hydrogenation of **1a** by using the retrieved **Pd8-H$_2$**. Reaction conditions: **1a** (0.5 mmol), biphenyl (0.25 mmol), retrieved **Pd8-H$_2$** (Pd: 0.5 mol%), diethyl ether (3 mL), room temperature (~25 °C), $H_2$ (1 atm), 18 h.

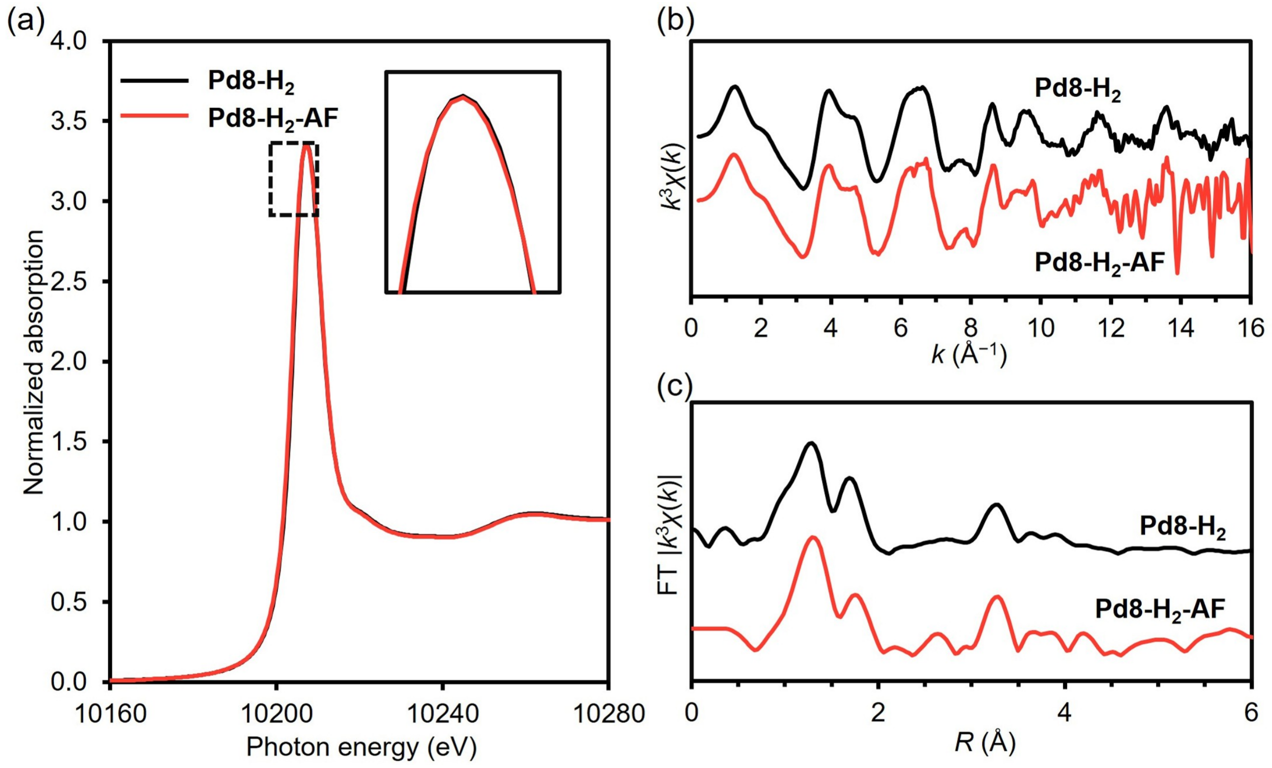


**Figure S10.** (a) W $L_3$-edgeXANES spectra, (b) $k^3$-weighted $k$-space, and (c) $R$-space EXAFS spectra of **Pd8-H$_2$** and retrieved **Pd8-H$_2$** after the hydrogenation of **1a** (**Pd8-H$_2$-AF**).

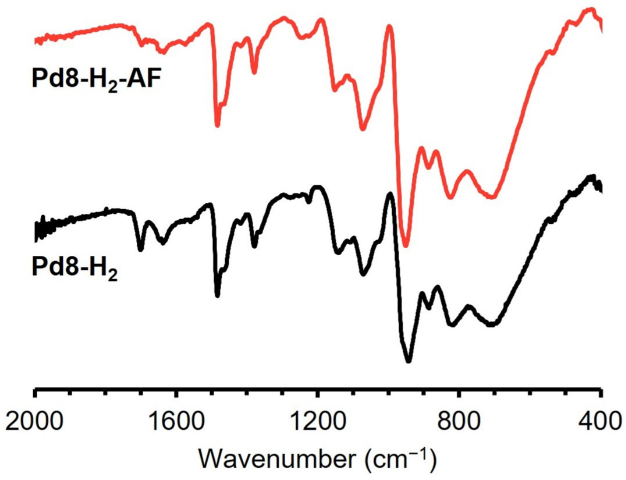


**Figure S11.** IR spectra of **Pd8-H$_2$** and retrieved **Pd8-H$_2$** the hydrogenation of **1a** (**Pd8-H$_2$-AF**).

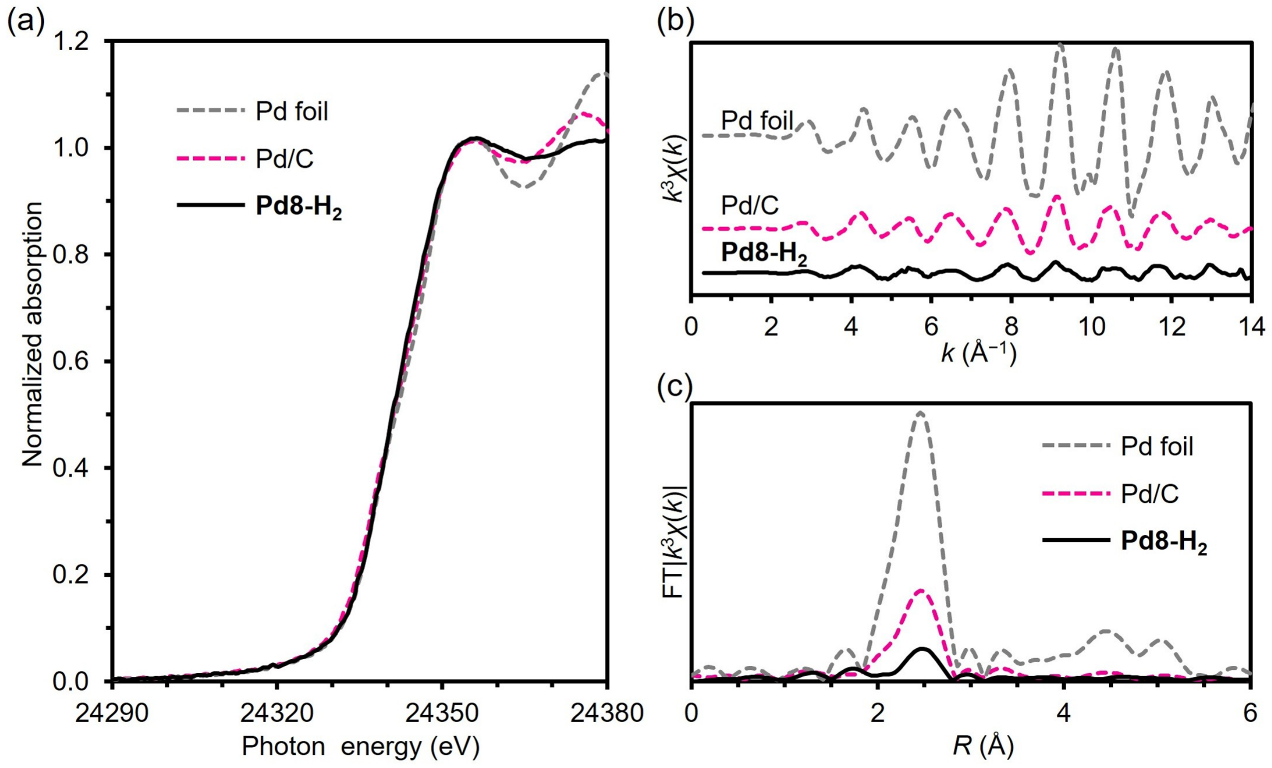


**Figure S12.** (a) Pd K-edge XANES spectra, (b) $k^3$-weighted *k*-space, and (c) *R*-space Pd K-edge EXAFS spectra of Pd foil, **Pd8-H$_2$**, and Pd/C.

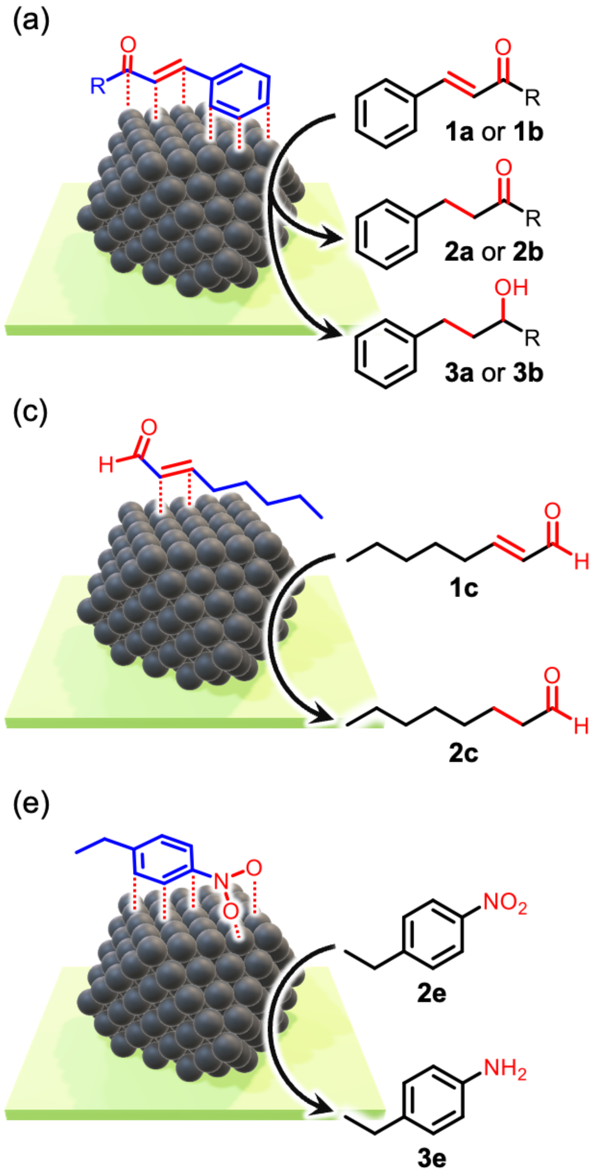


**Figure S13.** Hydrogenation of **1a**, **1b**, **1c**, and **2e** via different adsorption modes by (a,c,e) Pd/C and (b,d,f) **Pd8-H$_2$** and (R = H or Ph).

## References


[S1] S. Sasaki, K. Yonesato, N. Mizuno, K. Yamaguchi, K. Suzuki, *Inorg. Chem.* **2019**, *58*, 7722.

[S2] Rigaku O. D. CrysAlis PRO. Rigaku Oxford Diffraction Ltd, Yarnton, England (2018).

[S3] O. V. Dolomanov, L. J. Bourhis, R. J. Gildea, J. A. K. Howard, H. O. Puschmann, *J. Appl. Crystallogr.* **2009**, *42*, 339.

[S4] L. J. Farrugia, *J. Appl. Crystallogr.* **1999**, *32*, 837.

[S5] G. M. Sheldrick, *Acta Crystallogr. A* **2015**, *71*, 3.

[S6] a) G. M. Sheldrick, *Acta Crystallogr. A* **2008**, *64*, 112; b) G. M. Sheldrick, *Acta Crystallogr. C* **2015**, *71*, 3.

[S7] P. van der Sluis, A. L. Spek, *Acta Crystallogr. A* **1990**, *46*, 194.

[S8] N. E. Brese, M. O'Keeffe, *Acta Crystallogr. B* **1991**, *47*, 192.

[S9] *Radiat. Phys. Chem*. **2020**, *175*, 108270.

[S10] A. Ankudinov, B. Ravel, J. Rehr, S. Conradson, *Phys. Rev. B* **1998**, *58*, 7565.
